# Stabilizing regions of dominant pole placement for second order lead processes with time delay using filtered PID controllers

**DOI:** 10.1371/journal.pone.0304128

**Published:** 2024-06-25

**Authors:** Kaushik Halder, Saptarshi Das

**Affiliations:** 1 School of Computing and Electrical Engineering, Indian Institute of Technology, Mandi, Himachal Pradesh, India; 2 Centre for Environmental Mathematics, Faculty of Environment, Science and Economy, University of Exeter, Penryn, Cornwall, United Kingdom; 3 Institute for Data Science and Artificial Intelligence, University of Exeter, Exeter, Devon, United Kingdom; University of Shanghai for Science and Technology, CHINA

## Abstract

In order to handle second order lead processes with time delay, this paper provides a unique dominant pole placement based filtered PID controller design approach. This method does not require any finite term approximation like Pade to obtain the quasi-polynomial characteristic polynomial, arising due to the presence of the time delay term. The continuous time second order plus time delay systems with zero (SOPTDZ) are discretized using a pole-zero matching method with specified sampling time, where the transcendental exponential delay terms are converted into a finite number of poles. The pole-zero matching discretization approach with a predetermined sampling period is also used to discretize the continuous time filtered PID controller. As a result, it is not necessary to use any approximate discretization technique, such as Euler or Tustin, to derive the corresponding discrete time PID controller from its continuous time counterpart. The analytical expressions for discrete time dominant pole placement based filtered PID controllers are obtained using the coefficient matching approach, while two distinct kinds of non-dominant poles, namely all real and all complex conjugate, have been taken into consideration. The stabilizable region in the controller and design parameter space for the chosen class of linear second order time delay systems with lead is numerically approximated using the particle swarm optimization (PSO) based random search technique. The efficacy of the proposed method has been validated on a class of SOPTDZ systems including stable, integrating, unstable processes with minimum as well as non-minimum phase zeros.

## 1 Introduction

Many industrial processes contain large time delays which may cause a change in its dynamical characteristics in the form of oscillations or even reduce the stability of the control system [1]. Designing PID controllers for time delayed systems is difficult because the presence of time delay term makes the system to be of higher or infinite order. A finite order Pade approximation or infinite order Maclaurin series are often used for approximating the time delays.

### 1.1 Previous works on stability analysis of time delay systems

A number of researchers have attempted to design stabilizing controllers for handling systems with time delay. To handle time delay systems, for instance, smith predictor augmented PID controllers [2], PID controller based on internal model control (IMC) [3], Skogestad IMC (SIMC) [4, 5], approximate M-constrained integral gain optimization (AMIGO) [5, 6], linear matrix inequality (LMI) based output feedback [7], model predictive control (MPC) [8], and state feedback controllers [9, 10] etc. have been developed. These systems take either delay in the inputs [11] or in the states [9, 10] into consideration. Amongst these various controller design techniques for handling time delay systems, PID controllers have got special attention by the researchers in the past few decades due to its easy implementation, robust performance in the form of disturbance and noise rejection, set-point tracking performance etc. [12]. Finding the stability regions in the 2D controller parameter space for PI/PD [13, 14], or PID controllers [14–17] are the fundamental components of stability analysis for time-delayed systems. In order to obtain the stabilizable regions, several methods like the Hermite–Biehler theorem applicable to quasi-polynomials [16, 18], D-partitioning approach [19], Bode or Nyquist domain frequency response analysis [20], graphical methods [21, 22], stability boundary locus [14] etc. have been used. In [23–25], the 3D stability regions for delayed systems without lead and the controller without a low-pass filter were derived in the space of design criteria and PID controller parameters. Whereas 2D stability regions were found in the eigenvalue assignment of proportional, derivative, and filter parameter spaces in [26].

However, amongst these various approaches, very few researchers have obtained the stability region for the SOPTDZ systems. For example, 2D stability boundary of SOPTDZ process parameters have been obtained using Pontryagin’s criteria to design PID controller parameters in [27]. In [28, 29], 2D stability regions in PI and PID controller parameter space have been obtained using geometric approach for delayed processes with zero, respectively. In [30], the stability locus was used to generate stability regions for the SOPTDZ process in 2D PID controller parameter space by splitting the time delay term into real and imaginary parts. For a given value of proportional gain for the SOPTDZ process, the stability region was determined using a graphical technique in a 2D integral derivative plane in [22]. For time delay systems with an appropriate selection of controller parameters while satisfying designer’s specifications, the analysis of control loop performance is another crucial topic in addition to the stability analysis. However, the intrinsic time delay factor transforms the characteristic polynomial into a quasi-polynomial [16, 18]. Thus, it is challenging to analyze, ensure stability and control loop performance analytically by satisfying the given specifications of the control designer. For ensuring the specified control loop performances, dominant pole placement based PID controllers design can be used because of its ease of modification to accommodate the desired closed loop performance specifications e.g. natural frequency, damping ratio etc. [31–33] and these method has been adopted by earlier work like [23–25] for delayed systems without lead. The detailed literature survey on the design of dominant pole placement controller has been reported in [23–25, 32]. In addition, a technique for root distribution of quasi-polynomials using Pontryagin’s approach was developed to meet the requirements for assigning the dominant eigenvalues for retarded and neutral type time delay systems in [34]. Dominant pole placement-based PI-PD controller has been designed for handling arbitrary order systems with and without time delay, where the controller was discretized using Euler’s method in [35]. The work in [35], first parameterize the digital PID controllers by assigning dominant poles to the desired location. Then, the concept of Chebyshev polynomials was used to find the subset of digital PID controller parameters in which the remaining poles are located far away from the dominant pole pair. Finally, the obtained PID controller parameters were transformed into the PI-PD controller parameters by considering the closed-loop controller zero. Also, in [24, 25, 35, 36], the exponential delay terms were transformed into a finite number of poles in the discrete time *z*-plane by choosing an appropriate sampling time *T*_*s*_ in order to design the PID controller based on dominant pole placement. This transformation makes the characteristic equation rational.

In contrast to the approaches mentioned above, this paper employs a random search and optimization technique as an intelligent sampler to uncover the hidden pattern of stability region in the joint 7D filtered PID controller and dominant pole placement parameters for designing dominant pole placement based filtered PID controllers to handle SOPTDZ systems. In this case, the design parameter and controller parameter space might be multidimensional as opposed to earlier works when one controller gain was fixed in order to determine the others in a reduced dimensional space. Additionally, this work provides the dominant pole placement based filtered PID controller design methodology to stabilize the SOPTDZ systems without using Pade like finite order approximation of the delay. Here, it is worth noting that we are not comparing the control system performance with other PID tuning approaches like SIMC and AMIGO [4–6] since here the objective is different i.e. robust stabilization of delayed systems. In summary, this paper aims to show an analytical method of filtered PID controller design for a class of SOPTDZ systems i.e. stable, integrating, unstable SOPTDZ systems, where the main concern is to obtain a robust stability region.

### 1.2 Novelty of the present work

In order to handle fixed delayed SOPTDZ processes [37], this work proposes a novel filtered PID controller design method, based on dominant pole placement concept by extending related previous works on SOPTD process models without any lead or zero [23–25], delay free systems [32, 33] and first order plus time delay with zero (FOPTDZ) [38]. The characteristic equation becomes quasi-polynomial due to the inclusion of the time delay factor. But it can be converted into a rational equation by utilizing a finite order Pade approximation of the delay term [18, 23]. However, finite order Pade causes a rise in the number of poles and zeros in open loop systems, resulting in a higher order dynamics. In order to eliminate higher dimensional control problems due to finite order Pade approximation [18], here, the exponential delay term is transformed into finite number of poles. Using the proposed methodology of [36], the time delay term is transformed as *e*^−*Ls*^ = *z*^−*n*^ in discrete time domain, where the delay *L* is an integer multiple of the sampling time (*T*_*s*_) i.e. *n* = *L*/*T*_*s*_, $n\in Z_{+}$. With this conformal mapping of the time delay terms onto the discrete time domain, the appearance of unexpected open loop zeros due to finite term Pade approximation can also be avoided. In order to obtain *n* = *L*/*T*_*s*_, $n\in Z_{+}$, this paper considers the ideal scenario where the sampling time is chosen to be significantly less than the time delays and time constants of the open-loop systems i.e. *T*_*s*_ < < {*T*, *L*} which also relax the condition for approximating delays as *T*_*s*_ = *L*, as suggested in [36]. In contrast to the increased number of zeros and poles in the continuous time *s*-plane resulting from Pade approximation, it allows handling of finite number of poles in the discrete time complex *z*-plane.

In order to achieve the above goal, in this paper, the pole-zero matching approach with specified *T*_*s*_ has been used to discretize both the continuous time SOPTDZ process and PID controller with a derivative filter. However, it is not possible to obtain the discrete time equivalent DC gain of its continuous time version of the filtered PID controller using pole-zero discretization method. This is because in continuous time, the DC gain of the filtered PID controller will be infinite at *s* = 0. This problem can be overcome by using other approximate discretization method like Euler, Tustin method etc.[35, 39, 40] of discretization for obtaining the discrete equivalent PID controller as suggested in [24, 25]. However, these approximate discretization methods cannot ensure obtaining an equivalent discrete time filtered PID controller of its continuous time version. Therefore, using the pole-zero discretization method, the equivalent discrete time filtered PID controller transfer function can be obtained from its continuous time counterpart. For this, we have used *s* = *ε* where *ε* → 0 for transforming both zeros and poles of the system and PID controller, instead of using *s* = 0 due to the singularity problem. Then, the dominant pole placement based discrete time PID controller has been obtained using the coefficient matching method [41, 42] by satisfying the desired closed loop performance specifications, where the non-dominant poles can be either all real or all complex conjugates as reported in [23–25]. However, in this paper, we extend and generalize this concept for the SOPTDZ class of processes. According to the location of the pole placement parameter *m* i.e. *m* connected with both the real and imaginary part and only in the real part of the complex conjugate non-dominant poles, non-dominant complex conjugate poles are further divided into two kinds [23–25]. In order to control the continuous time SOPTDZ process as if the whole design has been completed in the continuous time domain, the obtained discrete time PID controller with a derivative filter is then transferred back to continuous time domain [23–25]. The main contributions of this paper are summarized as follows:

- Dominant pole placement based filtered PID controller has designed for handling SOPTDZ systems.
- The pole-zero matching method with specified sampling time is used to discretize the continuous time SOPTDZ systems, where the transcendental exponential delay terms are converted to a finite number of poles. The pole-zero matching discretization approach with a predetermined sampling period is also used to discretize the continuous time filtered PID controller. This approach allow us to avoid any finite term approximation like Pade for handling the delay term in the SOPTDZ systems and dicretization approximation like Euler, Tustin etc. for obtaining the discrete time PID controller from its continuous time counterpart.
- Three distinct analytical expressions for discrete time dominant pole placement based filtered PID controllers are obtained using the coefficient matching approach, while two different kinds of non-dominant poles such as all real and all complex conjugate have been considered.
- The stabilizable regions are obtained in the both controller and design parameter space for the chosen class of SOPTDZ processes using the PSO based random search technique.

## 2 Theoretical formulation

This section describes the transformation of continuous time SOPTDZ process and filtered PID controller in discrete time domain using pole-zero matching discretization approach [39]. Then, the dominant pole placement based filtered PID controller has been designed by the coefficient matching method [41, 42]. Now, SOPTDZ system in the continuous time domain can be considered as:

$$\begin{array}{c} G\left(s\right)=\frac{Y(s)}{U(s)}=\frac{K\left(\tau s+1\right)}{\left(T^{2}s^{2}+2\zeta_{ol}Ts+1\right)}e^{-Ls}. \end{array}$$ (1)

In (1), {*K*, *τ*, *T*, *ζ*_*ol*_, *L*} represents the DC gain, time constant of lead and lag, damping ratio, time delay of the system and *ω*_*ol*_ = 1/*T* represents the open-loop natural frequency. The SOPTDZ system (1) can also be represented as a neutral type delay differential equation (DDE) [43–45] as:

$$\begin{array}{c} T^{2}\frac{d^{2}y(t)}{dt^{2}}+2\zeta_{ol}T\frac{dy(t)}{dt}+y(t)=K\tau \frac{du(t-L)}{dt}+Ku(t-L). \end{array}$$ (2)

The characteristics of SOPTDZ systems can take the form of different filters based on the relative values of *T*, *ζ*_*ol*_ and *τ*. For example, system (2) can behave like a low-pass, band-pass and band-stop filter. Also, SOPTDZ system (2) is reduced to purely time delayed system i.e. *G*(*s*) = *Ke*^−*Ls*^ when *T* = *τ* = 0 which is tackled in [24].

The SOPTDZ system (1) can be controlled by the filtered PID controller:

$$\begin{array}{c} C(s)=K_{p}+\frac{K_{i}}{s}+\frac{K_{d}s}{1+T_{f}s}, \end{array}$$ (3)

where, {*K*_*p*_, *K*_*i*_, *K*_*d*_, *T*_*f*_} represents proportional, integral, derivative gains and derivative filter time constant respectively. Two poles and one zero of the open loop system (1) are located at:

$$\begin{array}{c} P_{p1,p2}=(-\frac{\zeta_{ol}}{T}\pm j\frac{1}{T}\sqrt{1-\zeta_{ol}^{2}}),Z_{p1}=-1/\tau , \end{array}$$ (4)

respectively.

Now, these continuous time poles and zero are mapped in the discrete time domain with the specified sampling time *T*_*s*_ as:

$$\begin{array}{c} {\tilde{P}}_{p1}=e^{P_{p1}T_{s}},{\tilde{P}}_{p2}=e^{P_{p2}T_{s}},{\tilde{Z}}_{p1}=e^{Z_{p1}T_{s}}. \end{array}$$ (5)

Similarly, two poles and two zeros of the continuous time filtered PID controller (3) are located at:

$$\begin{array}{c} P_{c1,c2}=\{0,-1/T_{f}\},\;Z_{c1,c2}=-\frac{\left(K_{p}+K_{i}T_{f}\right)}{2\left(K_{p}T_{f}+K_{d}\right)}\pm j\sqrt{\frac{K_{i}}{\left(K_{p}T_{f}+K_{d}\right)}-\frac{{\left(K_{p}+K_{i}T_{f}\right)}^{2}}{4{\left(K_{p}T_{f}+K_{d}\right)}^{2}}} \end{array}$$ (6)

respectively.

Using specified sampling time *T*_*s*_, the discretized form of filtered PID controller’s poles and zeros become:

$$\begin{array}{c} \begin{array}{c} {\tilde{P}}_{c1}=e^{P_{c1}T_{s}}=1\\ {\tilde{P}}_{c2}=e^{P_{c2}T_{s}}=e^{-T_{s}/T_{f}} \end{array}\}, \end{array}$$ (7)

and

$$\begin{array}{c} \begin{array}{c} {\tilde{Z}}_{c1,c2}=\{e^{Z_{c1}T_{s}},e^{Z_{c2}T_{s}}\}\\ \;=\text{exp}[\begin{array}{c} -\frac{(K_{p}+K_{i}T_{f})T_{s}}{2(K_{p}T_{f}+K_{d})}\pm jT_{s}\sqrt{\frac{K_{i}}{\left(K_{p}T_{f}+K_{d}\right)}-\frac{{\left(K_{p}+K_{i}T_{f}\right)}^{2}}{4{\left(K_{p}T_{f}+K_{d}\right)}^{2}}} \end{array}]\cdot \end{array} \end{array}$$ (8)

Now, using (5) and (7) and (8), the continuous time plants (1) and filtered PID controller (3) can be converted in the discrete time equivalent version and stated in following Lemmas.

*Lemma 1*: If the continuous time SOPTDZ process (1) is discretized using the pole-zero matching technique with the open loop poles and zero (5) and the sampling time *T*_*s*_, then the equivalent transfer function of (1) in discrete time will be:

$$\begin{array}{c} G(z)=\frac{{\tilde{K}}_{1}(z-{\tilde{Z}}_{p1})}{z^{n}(z-{\tilde{P}}_{p1})(z-{\tilde{P}}_{p2})}, \end{array}$$ (9)

where, $e^{-Ls}=z^{-n},\;n=L/T_{s}\in Z_{+}$ and

$$\begin{array}{c} \tilde{K}=\frac{K\tau (\varepsilon -Z_{p1})(e^{\varepsilon T_{s}}-{\tilde{P}}_{p1})(e^{\varepsilon T_{s}}-{\tilde{P}}_{p2})e^{\varepsilon (nT_{s}-L)}}{T^{2}(\varepsilon -P_{p1})(\varepsilon -P_{p2})(e^{\varepsilon T_{s}}-{\tilde{Z}}_{p1})}, \end{array}$$ (10)

represents the equivalent static gain in discrete time domain.

*Proof*: Using the continuous time open loop poles and zero *P*_*p*1_, *P*_*p*2_, *Z*_*p*1_, the system (1) can be represented as:

$$\begin{array}{c} G\left(s\right)=\frac{K\tau (s-Z_{p1})}{T^{2}(s-P_{p1})(s-P_{p2})}e^{-Ls}. \end{array}$$ (11)

The Eq (11) can be represented as (9) using the discrete time zero, poles (5) and sampling time *T*_*s*_. Now, using the pole-zero matching method by considering *s* = *ε* where *ε* → 0, and $z=e^{\varepsilon T_{s}}$ for the systems (11) and (9), the following hold:

$$\begin{array}{c} G_{1}\left(s\right){|}_{s=\varepsilon }=G_{1}(z){|}_{z=e^{\varepsilon T_{s}}}, \end{array}$$ (12)

which implies:

$$\begin{array}{c} \frac{K\tau (s-Z_{p1})}{T^{2}(s-P_{p1})(s-P_{p2})}e^{-Ls}{|}_{s=\varepsilon }=\frac{\tilde{K}(z-{\tilde{Z}}_{p1})}{z^{n}(z-{\tilde{P}}_{p1})(z-{\tilde{P}}_{p1})}{|}_{z=e^{\varepsilon T_{s}}}. \end{array}$$ (13)

By solving the Eq (13) analytically, (10) can be obtained.

*Lemma 2*: The equivalent discrete time filtered PID controller can be obtained from its continuous time version (3) using the pole-zero matching approach with the discrete time open loop zeroes (8), poles (7) and sampling time *T*_*s*_ as:

$$\begin{array}{c} C(z)=\frac{{\tilde{K}}_{C}(z-{\tilde{Z}}_{c1})(z-{\tilde{Z}}_{c2})}{\left(z-{\tilde{P}}_{c1})(z-{\tilde{P}}_{c2}\right)}, \end{array}$$ (14)

where,

$$\begin{array}{c} {\tilde{K}}_{C}=\frac{\begin{array}{c} \left((K_{p}T_{f}+K_{d})\varepsilon^{2}+(K_{p}+K_{i}T_{f})\varepsilon +K_{i})(e^{\varepsilon T_{s}}-{\tilde{P}}_{c1})(e^{\varepsilon T_{s}}-{\tilde{P}}_{c2}\right) \end{array}}{\varepsilon (1+T_{f}\varepsilon )(e^{\varepsilon T_{s}}-{\tilde{Z}}_{c1})(e^{\varepsilon T_{s}}-{\tilde{Z}}_{c2})}, \end{array}$$ (15)

is the static gain of the filtered PID controller in discrete time (14).

*Proof*: Similar to Lemma 1, we define *s* = *ε* with *ε* → 0 and $z=e^{\varepsilon T_{s}}$. Using (7), (8) and sampling time *T*_*s*_, the discrete equivalent form of (3) can be represented as (14). Now, the static gain can be obtained using the pole-zero matching method [39] using the (3) and (14) as:

$$\begin{array}{c} C(s){|}_{s=\varepsilon }=C(z){|}_{z=e^{\varepsilon T_{s}}}, \end{array}$$ (16)

which implies:

$$\begin{array}{c} \frac{(K_{p}T_{f}+K_{d})s^{2}+(K_{p}+K_{i}T_{f})s+K_{i}}{s(1+T_{f}s)}{|}_{s=\;\varepsilon }=\frac{{\tilde{K}}_{C}(z-{\tilde{Z}}_{c1})(z-{\tilde{Z}}_{c2})}{\left(z-{\tilde{P}}_{c1})(z-{\tilde{P}}_{c2}\right)}{|}_{z=e^{sT_{s}}}. \end{array}$$ (17)

By solving the above Eq (17) analytically yields:

$$\begin{array}{c} {\tilde{K}}_{C}=\frac{\begin{array}{c} \left((K_{p}T_{f}+K_{d})\varepsilon^{2}+(K_{p}+K_{i}T_{f})\varepsilon +K_{i})(e^{\varepsilon T_{s}}-{\tilde{P}}_{c1})(e^{\varepsilon T_{s}}-{\tilde{P}}_{c2}\right) \end{array}}{\varepsilon (1+T_{f}\varepsilon )(e^{\varepsilon T_{s}}-{\tilde{Z}}_{c1})(e^{\varepsilon T_{s}}-{\tilde{Z}}_{c2})}. \end{array}$$ (18)

## 3 Filtered PID controller design for SOPTDZ process based on dominant pole placement

### 3.1 The generic non-dominant pole placement set-up

In this section, the dominant pole placement based filtered PID controller for the SOPTDZ process (1) has been designed using the coefficient matching method. Two dominant and separate non-dominant pole types, namely all real and all complex conjugate, have been taken into consideration in order to get the analytical expressions of the four PID controller parameters {*K*_*p*_, *K*_*i*_, *K*_*d*_, *T*_*f*_} using the coefficient matching approach, as studied in [23–25]. Again, there are two different types of all complex conjugate non-dominant poles, depending on the pole placement parameter’s *m* location in the poles, i.e., *m* is associated solely with the real part or *m* is connected with both the real and imaginary parts. As a result, three distinct non-dominant pole types can be used to derive a total of three analytical formulations for the system (1). Using the designer’s closed-loop specifications {*m*, *ζ*_*cl*_, *ω*_*cl*_}, where $m\in R_{+}$ and $\{\zeta_{cl},\omega_{cl}\}\in R$, the continuous time complex conjugate dominant poles and all real non-dominant poles can be represented as:

$$\begin{array}{c} P_{1,2}^{d}=-\zeta_{cl}\omega_{cl}\pm j\omega_{cl}\sqrt{1-\zeta_{cl}^{2}},\;P_{3}^{nd}=-m\zeta_{cl}\omega_{cl}. \end{array}$$ (19)

Once more, we denote the two distinct types of complex conjugate non-dominant poles in continuous time where *m* is associated with the both real and imaginary part and *m* is in real part only as:

$$\begin{array}{c} \begin{array}{c} P_{4,5}^{nd}=m(-\zeta_{cl}\omega_{cl}\pm j\omega_{cl}\sqrt{1-\zeta_{cl}^{2}}),\;\\ P_{6,7}^{nd}=(-m\zeta_{cl}\omega_{cl}\pm j\omega_{cl}\sqrt{1-\zeta_{cl}^{2}}) \end{array}\}, \end{array}$$ (20)

respectively.

Now, mapping all the continuous time dominant and non-dominant poles in (19), (20) with the sampling time *T*_*s*_ in discrete time yields:

$$\begin{array}{c} {\tilde{P}}_{1}^{d}=e^{P_{1}^{d}T_{s}},{\tilde{P}}_{2}^{d}=e^{P_{2}^{d}T_{s}},{\tilde{P}}_{3}^{nd}=e^{P_{3}^{nd}T_{s}}, \end{array}$$ (21)

and

$$\begin{array}{c} \begin{array}{c} {\tilde{P}}_{4}^{nd}=e^{P_{4}^{nd}T_{s}},{\tilde{P}}_{5}^{nd}=e^{P_{5}^{nd}T_{s}},\\ {\tilde{P}}_{6}^{nd}=e^{P_{6}^{nd}T_{s}},{\tilde{P}}_{7}^{nd}=e^{P_{7}^{nd}T_{s}} \end{array}\}. \end{array}$$ (22)

The four PID controller parameters {*K*_*p*_, *K*_*i*_, *K*_*d*_, *T*_*f*_} for stabilizing the SOPTDZ system (1) are now determined using these discretized dominant and non-dominant poles (21) and (22), as explained in the next sub-sections.

### 3.2 All real non-dominant poles

Three sets of algebraic expressions are derived from three different non-dominant pole types (21), (22) and characterized by the following theorems in order to guarantee dominant pole placement for the SOPTDZ process (1) with the filtered PID controller.

*Theorem 3*: If the non-dominant poles are all real in nature (21), then the following simultaneous nonlinear implicit equations need to be solved in order to obtain the PID controller parameters {*K*_*p*_, *K*_*i*_, *K*_*d*_, *T*_*f*_} for achieving dominant pole placement with the filtered PID controller for the system (1):

$$\begin{array}{c} \begin{array}{c} z^{0}:\;\psi_{1}=\tilde{K}{\tilde{K}}_{C}{\tilde{Z}}_{c1}{\tilde{Z}}_{c2}{\tilde{Z}}_{p1}+A_{0}=0,\\ z^{1}:\;\psi_{2}=\tilde{K}{\tilde{K}}_{C}({\tilde{Z}}_{c1}{\tilde{Z}}_{c2}+{\tilde{Z}}_{c1}{\tilde{Z}}_{p1}+{\tilde{Z}}_{c2}{\tilde{Z}}_{p1})-A_{1}=0,\\ z^{2}:\;\psi_{3}=\tilde{K}{\tilde{K}}_{C}({\tilde{Z}}_{c1}+{\tilde{Z}}_{c2}+{\tilde{Z}}_{p1})+A_{2}=0,\\ z^{3}:\;\psi_{4}=\tilde{K}{\tilde{K}}_{C}-A_{3}=0,\\ z^{n}:\;\psi_{5}={\tilde{P}}_{c1}{\tilde{P}}_{c2}{\tilde{P}}_{p1}{\tilde{P}}_{p2}-A_{n}=0,\\ z^{n+1}:\;\psi_{6}={\tilde{P}}_{p1}{\tilde{P}}_{p2}{\tilde{P}}_{c1}+{\tilde{P}}_{p1}{\tilde{P}}_{p2}{\tilde{P}}_{c2}+{\tilde{P}}_{c1}{\tilde{P}}_{c2}{\tilde{P}}_{p1}\\ \;\;\;\;+{\tilde{P}}_{c1}{\tilde{P}}_{c2}{\tilde{P}}_{p2}+A_{n+1}=0,\\ z^{n+2}:\;\psi_{7}={\tilde{P}}_{p1}{\tilde{P}}_{p2}+{\tilde{P}}_{p1}{\tilde{P}}_{c1}+{\tilde{P}}_{p1}{\tilde{P}}_{c2}+{\tilde{P}}_{p2}{\tilde{P}}_{c1}\\ \;\;\;\;+{\tilde{P}}_{p2}{\tilde{P}}_{c2}+{\tilde{P}}_{c1}{\tilde{P}}_{c2}-A_{n+2}=0,\\ z^{n+3}:\;\psi_{8}={\tilde{P}}_{c1}+{\tilde{P}}_{c2}+{\tilde{P}}_{p1}+{\tilde{P}}_{p2}+A_{n+3}=0. \end{array} \end{array}$$ (23)

In (23), ${\tilde{K}}_{C}=f(K_{p},K_{i},K_{d},T_{f})$, ${\tilde{P}}_{c2}=f(T_{f})$ and ${\tilde{Z}}_{c1,c2}=f(K_{p},K_{i},K_{d},T_{f})$ as in (15), (7) and (8) respectively.

*Proof*: Using (9) and (15) from *Lemma 1* and *Lemma 2*, the characteristic equation in closed loop can be represented as:

$$\delta \left(z,K_{p},K_{i},K_{d},T_{f}\right)≜=1+C\left(z\right)G_{1}\left(z\right)≜0.$$ (24)

This implies,

$$\begin{array}{c} 1+\frac{{\tilde{K}}_{C}{\tilde{K}}_{1}(z-{\tilde{Z}}_{c1})(z-{\tilde{Z}}_{c2})(z-{\tilde{Z}}_{p1})}{z^{n}(z-{\tilde{P}}_{c1})(z-{\tilde{P}}_{c2})(z-{\tilde{P}}_{p1})(z-{\tilde{P}}_{p2})}=0. \end{array}$$ (25)

The Eq (25) yields:

$$\begin{array}{c} \begin{array}{c} z^{n+4}[1]\\ +z^{n+3}[-({\tilde{P}}_{p1}+{\tilde{P}}_{p2}+{\tilde{P}}_{c1}+{\tilde{P}}_{c2})]\\ +z^{n+2}[\begin{array}{c} {\tilde{P}}_{p1}{\tilde{P}}_{p2}+{\tilde{P}}_{p2}{\tilde{P}}_{c1}+{\tilde{P}}_{p2}{\tilde{P}}_{c2}+{\tilde{P}}_{p1}{\tilde{P}}_{c1}+{\tilde{P}}_{p1}{\tilde{P}}_{c2}+{\tilde{P}}_{c1}{\tilde{P}}_{c2} \end{array}]\\ +z^{n+1}[-(\begin{array}{c} {\tilde{P}}_{p1}{\tilde{P}}_{p2}{\tilde{P}}_{c1}+{\tilde{P}}_{p1}{\tilde{P}}_{p2}{\tilde{P}}_{c2}+{\tilde{P}}_{p2}{\tilde{P}}_{c1}{\tilde{P}}_{c2}+{\tilde{P}}_{p1}{\tilde{P}}_{c1}{\tilde{P}}_{c2} \end{array})]\\ +z^{n}[{\tilde{P}}_{p1}{\tilde{P}}_{p2}{\tilde{P}}_{c1}{\tilde{P}}_{c2}]\\ +z^{3}[{\tilde{K}}_{C}\tilde{K}]\\ +z^{2}[-{\tilde{K}}_{C}\tilde{K}({\tilde{Z}}_{c1}+{\tilde{Z}}_{c2}+{\tilde{Z}}_{p1})]\\ +z[{\tilde{K}}_{C}\tilde{K}({\tilde{Z}}_{p1}{\tilde{Z}}_{c1}+{\tilde{Z}}_{p1}{\tilde{Z}}_{c2}+{\tilde{Z}}_{c1}{\tilde{Z}}_{c2})]\\ +z^{0}[-{\tilde{K}}_{C}\tilde{K}{\tilde{Z}}_{p1}{\tilde{Z}}_{c1}{\tilde{Z}}_{c2}]=0. \end{array} \end{array}$$ (26)

The desired characteristic polynomial will be of (*n* + 4)^*th*^ order as the order of the characteristic polynomial presented in (26) is (*n* + 4). Now, the desired characteristic equation can be expressed considering the two dominant poles and the other poles as real non-dominant pole type (21) as follows:

$$\begin{array}{c} \Delta \left(z,m,\zeta_{cl},\omega_{cl},T_{s}\right)=\left(z-{\tilde{P}}_{1}^{d}\right)\left(z-{\tilde{P}}_{2}^{d}\right){\left(z-{\tilde{P}}_{3}^{nd}\right)}^{n+2}=0. \end{array}$$ (27)

This implies,

$$\begin{array}{c} \begin{array}{c} z^{n+4}[\left(\begin{array}{c} n+2\\ 0 \end{array}\right){\left(-{\tilde{P}}_{3}^{nd}\right)}^{0}]+\\ z^{n+3}[\begin{array}{c} \left(\begin{array}{c} n+2\\ 1 \end{array}\right)\left(-{\tilde{P}}_{3}^{nd}\right)-\left(\begin{array}{c} n+2\\ 0 \end{array}\right){\left(-{\tilde{P}}_{3}^{nd}\right)}^{0}\left({\tilde{P}}_{d1}+{\tilde{P}}_{d2}\right) \end{array}]+\\ z^{n+2}[\begin{array}{c} \left(\begin{array}{c} n+2\\ 2 \end{array}\right){\left(-{\tilde{P}}_{3}^{nd}\right)}^{2}-\left(\begin{array}{c} n+2\\ 1 \end{array}\right)\left(-{\tilde{P}}_{3}^{nd}\right)\left({\tilde{P}}_{d1}+{\tilde{P}}_{d2}\right)\\ +\left(\begin{array}{c} n+2\\ 0 \end{array}\right){\left(-{\tilde{P}}_{3}^{nd}\right)}^{0}{\tilde{P}}_{d1}{\tilde{P}}_{d2} \end{array}]+\\ z^{n+1}[\begin{array}{c} \left(\begin{array}{c} n+2\\ 3 \end{array}\right){\left(-{\tilde{P}}_{3}^{nd}\right)}^{3}-\left(\begin{array}{c} n+2\\ 2 \end{array}\right){\left(-{\tilde{P}}_{3}^{nd}\right)}^{2}\left({\tilde{P}}_{d1}+{\tilde{P}}_{d2}\right)\\ +\left(\begin{array}{c} n+2\\ 1 \end{array}\right)\left(-{\tilde{P}}_{3}^{nd}\right){\tilde{P}}_{d1}{\tilde{P}}_{d2} \end{array}]+ \end{array} \end{array}$$

$$\begin{array}{c} \begin{array}{c} z^{n}[\begin{array}{c} \left(\begin{array}{c} n+2\\ 4 \end{array}\right){\left(-{\tilde{P}}_{3}^{nd}\right)}^{4}-\left(\begin{array}{c} n+2\\ 3 \end{array}\right){\left(-{\tilde{P}}_{3}^{nd}\right)}^{3}\left({\tilde{P}}_{d1}+{\tilde{P}}_{d2}\right)\\ +\left(\begin{array}{c} n+2\\ 2 \end{array}\right){\left(-{\tilde{P}}_{3}^{nd}\right)}^{2}{\tilde{P}}_{d1}{\tilde{P}}_{d2} \end{array}]+\cdots +\\ z^{3}[\begin{array}{c} \left(\begin{array}{c} n+2\\ n+1 \end{array}\right){\left(-{\tilde{P}}_{3}^{nd}\right)}^{n+1}-\left(\begin{array}{c} n+2\\ n \end{array}\right){\left(-{\tilde{P}}_{3}^{nd}\right)}^{n}\left({\tilde{P}}_{d1}+{\tilde{P}}_{d2}\right)\\ +\left(\begin{array}{c} n+2\\ n-1 \end{array}\right){\left(-{\tilde{P}}_{3}^{nd}\right)}^{n-1}{\tilde{P}}_{d1}{\tilde{P}}_{d2} \end{array}]+\\ z^{2}[\begin{array}{c} \left(\begin{array}{c} n+2\\ n+2 \end{array}\right){\left(-{\tilde{P}}_{3}^{nd}\right)}^{n+2}-\left(\begin{array}{c} n+2\\ n+1 \end{array}\right){\left(-{\tilde{P}}_{3}^{nd}\right)}^{n+1}\left({\tilde{P}}_{d1}+{\tilde{P}}_{d2}\right)\\ +\left(\begin{array}{c} n+2\\ n \end{array}\right){\left(-{\tilde{P}}_{3}^{nd}\right)}^{n}{\tilde{P}}_{d1}{\tilde{P}}_{d2} \end{array}]+\\ z^{1}[\begin{array}{c} -\left(\begin{array}{c} n+2\\ n+2 \end{array}\right){\left(-{\tilde{P}}_{3}^{nd}\right)}^{n+2}\left({\tilde{P}}_{d1}+{\tilde{P}}_{d2}\right)+\\ \left(\begin{array}{c} n+2\\ n+1 \end{array}\right){\left(-{\tilde{P}}_{3}^{nd}\right)}^{n+1}{\tilde{P}}_{d1}{\tilde{P}}_{d2} \end{array}]+\\ z^{0}[\left(\begin{array}{c} n+2\\ n+2 \end{array}\right){\left(-{\tilde{P}}_{3}^{nd}\right)}^{n+2}{\tilde{P}}_{d1}{\tilde{P}}_{d2}]=0, \end{array} \end{array}$$ (28)

where, (nr)=Cnr=n!r!(n-r)! represents the binomial coefficient.

The Eq (28) can also be written as:

$$\begin{array}{c} \begin{array}{c} \Delta (z,m,\zeta_{cl},\omega_{cl},T_{s})=A_{n+4}z^{n+4}+A_{n+3}z^{n+3}+\cdots +A_{2}z^{2}+A_{1}z^{1}+A_{0}z^{0}=0, \end{array} \end{array}$$ (29)

where, *A*_*j*_, *j* = {(*n*+ 4), (*n*+ 2), ⋯, 2, 1, 0} are the coefficients of the characteristic Eq (28) which can be calculated using the desired specifications of the closed-loop system {*m*, *ζ*_*cl*_, *ω*_*cl*_}. Now, matching the coefficients of (26) and (29) yields (23).

The coefficients of *z*^0^ to *z*^3^ are used for obtaining the three unknown parameters {*K*_*p*_, *K*_*i*_, *K*_*d*_} or the gains of PID controller while the derivative filter gain *T*_*f*_ can be obtained using the coefficients of *z*^*n*^ to *z*^*n*+2^.

### 3.3 All complex conjugate non-dominant poles

In this section, we show that the pole placement parameter (*m*) can be attached to either only real part or both real and imaginary parts of the complex conjugate non-dominant poles, yielding to the following two theorems to find the controller parameters by simultaneous equation solving.

*Theorem 4*: Given that the pole placement parameter (*m*) is connected to both the real and imaginary parts of all complex conjugate non-dominant poles, the following nonlinear implicit equations must be solved for determining the gains {*K*_*p*_, *K*_*i*_, *K*_*d*_, *T*_*f*_} of the filtered PID controller in order to achieve dominant pole placement of SOPTDZ system (1):

$$\begin{array}{c} \begin{array}{c} z^{0}:\;\psi_{1}=\tilde{K}{\tilde{K}}_{C}{\tilde{Z}}_{c1}{\tilde{Z}}_{c2}{\tilde{Z}}_{p1}+{\tilde{A}}_{0}=0,\\ z^{1}:\;\psi_{2}=\tilde{K}{\tilde{K}}_{C}({\tilde{Z}}_{c1}{\tilde{Z}}_{c2}+{\tilde{Z}}_{c1}{\tilde{Z}}_{p1}+{\tilde{Z}}_{c2}{\tilde{Z}}_{p1})-{\tilde{A}}_{1}=0,\\ z^{2}:\;\psi_{3}=\tilde{K}{\tilde{K}}_{C}({\tilde{Z}}_{c1}+{\tilde{Z}}_{c2}+{\tilde{Z}}_{p1})+{\tilde{A}}_{2}=0,\\ z^{3}:\;\psi_{4}=\tilde{K}{\tilde{K}}_{C}-{\tilde{A}}_{3}=0,\\ z^{n}:\;\psi_{5}={\tilde{P}}_{c1}{\tilde{P}}_{c2}{\tilde{P}}_{p1}{\tilde{P}}_{p2}-{\tilde{A}}_{n}=0,\\ z^{n+1}:\;\psi_{6}={\tilde{P}}_{p1}{\tilde{P}}_{p2}{\tilde{P}}_{c1}+{\tilde{P}}_{p1}{\tilde{P}}_{p2}{\tilde{P}}_{c2}+{\tilde{P}}_{c1}{\tilde{P}}_{c2}{\tilde{P}}_{p1}+{\tilde{P}}_{c1}{\tilde{P}}_{c2}{\tilde{P}}_{p2}+{\tilde{A}}_{n+1}=0,\\ z^{n+2}:\;\psi_{7}={\tilde{P}}_{p1}{\tilde{P}}_{p2}+{\tilde{P}}_{p1}{\tilde{P}}_{c1}+{\tilde{P}}_{p1}{\tilde{P}}_{c2}+{\tilde{P}}_{p2}{\tilde{P}}_{c1}+{\tilde{P}}_{p2}{\tilde{P}}_{c2}+{\tilde{P}}_{c1}{\tilde{P}}_{c2}-{\tilde{A}}_{n+2}=0,\\ z^{n+3}:\;\psi_{8}={\tilde{P}}_{c1}+{\tilde{P}}_{c2}+{\tilde{P}}_{p1}+{\tilde{P}}_{p2}+{\tilde{A}}_{n+3}=0. \end{array} \end{array}$$ (30)

*Proof*: The (*n* + 4)^*th*^ order closed loop characteristic polynomial with two dominant {${\tilde{P}}_{1}^{d},{\tilde{P}}_{2}^{d}$} (21) and all complex conjugate non-dominant poles {${\tilde{P}}_{4}^{nd},{\tilde{P}}_{5}^{nd}$} (22) can be written as:

$$\begin{array}{c} \Delta \left(z,m,\zeta_{cl},\omega_{cl},T_{s}\right)=\left(z-{\tilde{P}}_{1}^{d}\right)\left(z-{\tilde{P}}_{2}^{d}\right){\left(z-{\tilde{P}}_{4}^{nd}\right)}^{\frac{n+2}{2}}{\left(z-{\tilde{P}}_{5}^{nd}\right)}^{\frac{n+2}{2}}=0. \end{array}$$ (31)

Expanding (31) using binomial expansion yields:

$$\begin{array}{c} \begin{array}{c} z^{n+4}[\left(\begin{array}{c} \frac{n+2}{2}\\ 0 \end{array}\right)\left(\begin{array}{c} \frac{n+2}{2}\\ 0 \end{array}\right){\left(-{\tilde{P}}_{4}^{nd}\right)}^{0}{\left(-{\tilde{P}}_{5}^{nd}\right)}^{0}]+\\ z^{n+3}[\begin{array}{c} \left(\begin{array}{c} \frac{n+2}{2}\\ 0 \end{array}\right)\left(\begin{array}{c} \frac{n+2}{2}\\ 1 \end{array}\right)\left(\begin{array}{c} {\left(-{\tilde{P}}_{4}^{nd}\right)}^{0}\left(-{\tilde{P}}_{5}^{nd}\right)+\\ \left(-{\tilde{P}}_{4}^{nd}\right){\left(-{\tilde{P}}_{5}^{nd}\right)}^{0} \end{array}\right)\\ -\left(\begin{array}{c} \frac{n+2}{2}\\ 0 \end{array}\right)\left(\begin{array}{c} \frac{n+2}{2}\\ 0 \end{array}\right)\left(\begin{array}{c} {\left(-{\tilde{P}}_{4}^{nd}\right)}^{0}{\left(-{\tilde{P}}_{5}^{nd}\right)}^{0}\\ \times \left({\tilde{P}}_{1}^{d}+{\tilde{P}}_{2}^{d}\right) \end{array}\right) \end{array}]+ \end{array} \end{array}$$

$$\begin{array}{c} \begin{array}{c} z^{n+2}[\begin{array}{c} \left(\begin{array}{c} \frac{n+2}{2}\\ 0 \end{array}\right)\left(\begin{array}{c} \frac{n+2}{2}\\ 2 \end{array}\right)\left(\begin{array}{c} {\left(-{\tilde{P}}_{4}^{nd}\right)}^{0}{\left(-{\tilde{P}}_{5}^{nd}\right)}^{2}+\\ {\left(-{\tilde{P}}_{4}^{nd}\right)}^{2}{\left(-{\tilde{P}}_{5}^{nd}\right)}^{0} \end{array}\right)\\ -\left(\begin{array}{c} \frac{n+2}{2}\\ 0 \end{array}\right)\left(\begin{array}{c} \frac{n+2}{2}\\ 1 \end{array}\right)\left({\tilde{P}}_{1}^{d}+{\tilde{P}}_{2}^{d}\right)\times \\ \left({\left(-{\tilde{P}}_{4}^{nd}\right)}^{0}\left(-{\tilde{P}}_{5}^{nd}\right)+\left(-{\tilde{P}}_{4}^{nd}\right){\left(-{\tilde{P}}_{5}^{nd}\right)}^{0}\right)\\ +{\left(\begin{array}{c} \frac{n+2}{2}\\ 0 \end{array}\right)}^{2}{\left(-{\tilde{P}}_{4}^{nd}\right)}^{0}{\left(-{\tilde{P}}_{5}^{nd}\right)}^{0}\\ \times {\tilde{P}}_{1}^{d}{\tilde{P}}_{2}^{d}+{\left(\begin{array}{c} \frac{n+2}{2}\\ 1 \end{array}\right)}^{2}\left(-{\tilde{P}}_{4}^{nd}\right)\left(-{\tilde{P}}_{5}^{nd}\right) \end{array}]+\\ z^{n+1}[\begin{array}{c} \left(\begin{array}{c} \frac{n+2}{2}\\ 0 \end{array}\right)\left(\begin{array}{c} \frac{n+2}{2}\\ 3 \end{array}\right)\left(\begin{array}{c} {\left(-{\tilde{P}}_{4}^{nd}\right)}^{0}{\left(-{\tilde{P}}_{5}^{nd}\right)}^{3}\\ +{\left(-{\tilde{P}}_{4}^{nd}\right)}^{3}{\left(-{\tilde{P}}_{5}^{nd}\right)}^{0} \end{array}\right)\\ -\left(\begin{array}{c} \frac{n+2}{2}\\ 0 \end{array}\right)\left(\begin{array}{c} \frac{n+2}{2}\\ 2 \end{array}\right)\left(\begin{array}{c} {\left(-{\tilde{P}}_{4}^{nd}\right)}^{0}{\left(-{\tilde{P}}_{5}^{nd}\right)}^{2}\\ +{\left(-{\tilde{P}}_{4}^{nd}\right)}^{2}{\left(-{\tilde{P}}_{5}^{nd}\right)}^{0} \end{array}\right)\left({\tilde{P}}_{1}^{d}+{\tilde{P}}_{2}^{d}\right)\\ +\left(\begin{array}{c} \frac{n+2}{2}\\ 0 \end{array}\right)\left(\begin{array}{c} \frac{n+2}{2}\\ 1 \end{array}\right){\tilde{P}}_{1}^{d}{\tilde{P}}_{2}^{d}\\ \times \left({\left(-{\tilde{P}}_{4}^{nd}\right)}^{0}\left(-{\tilde{P}}_{5}^{nd}\right)+\left(-{\tilde{P}}_{4}^{nd}\right){\left(-{\tilde{P}}_{5}^{nd}\right)}^{0}\right)\\ +\left(\begin{array}{c} \frac{n+2}{2}\\ 1 \end{array}\right)\left(\begin{array}{c} \frac{n+2}{2}\\ 2 \end{array}\right)\left(\begin{array}{c} \left(-{\tilde{P}}_{4}^{nd}\right){\left(-{\tilde{P}}_{5}^{nd}\right)}^{2}\\ +{\left(-{\tilde{P}}_{4}^{nd}\right)}^{2}\left(-{\tilde{P}}_{5}^{nd}\right) \end{array}\right)\\ -{\left(\begin{array}{c} \frac{n+2}{2}\\ 1 \end{array}\right)}^{2}\left(-{\tilde{P}}_{4}^{nd}\right)\left(-{\tilde{P}}_{5}^{nd}\right)\left({\tilde{P}}_{1}^{d}+{\tilde{P}}_{2}^{d}\right) \end{array}]+\\ z^{n}[\begin{array}{c} \left(\begin{array}{c} \frac{n+2}{2}\\ 0 \end{array}\right)\left(\begin{array}{c} \frac{n+2}{2}\\ 4 \end{array}\right)\left(\begin{array}{c} {\left(-{\tilde{P}}_{4}^{nd}\right)}^{0}{\left(-{\tilde{P}}_{5}^{nd}\right)}^{4}\\ +{\left(-{\tilde{P}}_{4}^{nd}\right)}^{4}{\left(-{\tilde{P}}_{5}^{nd}\right)}^{0} \end{array}\right)\\ +\left(\begin{array}{c} \frac{n+2}{2}\\ 1 \end{array}\right)\left(\begin{array}{c} \frac{n+2}{2}\\ 3 \end{array}\right)\left(\begin{array}{c} \left(-{\tilde{P}}_{4}^{nd}\right){\left(-{\tilde{P}}_{5}^{nd}\right)}^{3}\\ +{\left(-{\tilde{P}}_{4}^{nd}\right)}^{3}\left(-{\tilde{P}}_{5}^{nd}\right) \end{array}\right)\\ -\left(\begin{array}{c} \frac{n+2}{2}\\ 0 \end{array}\right)\left(\begin{array}{c} \frac{n+2}{2}\\ 3 \end{array}\right)\left(\begin{array}{c} {\left(-{\tilde{P}}_{4}^{nd}\right)}^{0}{\left(-{\tilde{P}}_{5}^{nd}\right)}^{3}\\ +{\left(-{\tilde{P}}_{4}^{nd}\right)}^{3}{\left(-{\tilde{P}}_{5}^{nd}\right)}^{0} \end{array}\right)\left({\tilde{P}}_{1}^{d}+{\tilde{P}}_{2}^{d}\right)\\ -\left(\begin{array}{c} \frac{n+2}{2}\\ 1 \end{array}\right)\left(\begin{array}{c} \frac{n+2}{2}\\ 2 \end{array}\right)\left(\begin{array}{c} \left(-{\tilde{P}}_{4}^{nd}\right){\left(-{\tilde{P}}_{5}^{nd}\right)}^{2}\\ +{\left(-{\tilde{P}}_{4}^{nd}\right)}^{2}\left(-{\tilde{P}}_{5}^{nd}\right) \end{array}\right)\\ \times \left({\tilde{P}}_{1}^{d}+{\tilde{P}}_{2}^{d}\right)+\left(\begin{array}{c} \frac{n+2}{2}\\ 0 \end{array}\right)\left(\begin{array}{c} \frac{n+2}{2}\\ 2 \end{array}\right){\tilde{P}}_{1}^{d}{\tilde{P}}_{2}^{d}\\ \times \left(\begin{array}{c} {\left(-{\tilde{P}}_{4}^{nd}\right)}^{0}{\left(-{\tilde{P}}_{5}^{nd}\right)}^{2}\\ +{\left(-{\tilde{P}}_{4}^{nd}\right)}^{2}{\left(-{\tilde{P}}_{5}^{nd}\right)}^{0} \end{array}\right)+{\left(\begin{array}{c} \frac{n+2}{2}\\ 2 \end{array}\right)}^{2}{\left(-{\tilde{P}}_{4}^{nd}\right)}^{2}{\left(-{\tilde{P}}_{5}^{nd}\right)}^{2}\\ +{\left(\begin{array}{c} \frac{n+2}{2}\\ 1 \end{array}\right)}^{2}\left(-{\tilde{P}}_{4}^{nd}\right)\left(-{\tilde{P}}_{5}^{nd}\right){\tilde{P}}_{1}^{d}{\tilde{P}}_{2}^{d} \end{array}]\\ +\cdots + \end{array} \end{array}$$

$$\begin{array}{c} \begin{array}{c} z^{3}[\begin{array}{c} -\left({\tilde{P}}_{1}^{d}+{\tilde{P}}_{2}^{d}\right)\times \\ \left(\begin{array}{c} \left(\begin{array}{c} \frac{n+2}{2}\\ \frac{n-2}{2} \end{array}\right)\left(\begin{array}{c} \frac{n+2}{2}\\ \frac{n+2}{2} \end{array}\right)\times \\ \left(\begin{array}{c} {\left(-{\tilde{P}}_{4}^{nd}\right)}^{\frac{n-2}{2}}{\left(-{\tilde{P}}_{5}^{nd}\right)}^{\frac{n+2}{2}}+{\left(-{\tilde{P}}_{4}^{nd}\right)}^{\frac{n+2}{2}}{\left(-{\tilde{P}}_{5}^{nd}\right)}^{\frac{n-2}{2}} \end{array}\right)+\\ {\left(\begin{array}{c} \frac{n+2}{2}\\ \frac{n}{2} \end{array}\right)}^{2}{\left(-{\tilde{P}}_{4}^{nd}\right)}^{\frac{n}{2}}{\left(-{\tilde{P}}_{5}^{nd}\right)}^{\frac{n}{2}} \end{array}\right)\\ +\left(\begin{array}{c} \frac{n+2}{2}\\ \frac{n+2}{2} \end{array}\right)\left(\begin{array}{c} \frac{n+2}{2}\\ \frac{n}{2} \end{array}\right)\left(\begin{array}{c} {\left(-{\tilde{P}}_{4}^{nd}\right)}^{\frac{n}{2}}{\left(-{\tilde{P}}_{5}^{nd}\right)}^{\frac{n+2}{2}}\\ +{\left(-{\tilde{P}}_{4}^{nd}\right)}^{\frac{n+2}{2}}{\left(-{\tilde{P}}_{5}^{nd}\right)}^{\frac{n}{2}} \end{array}\right)+\\ {\tilde{P}}_{1}^{d}{\tilde{P}}_{2}^{d}\left(\begin{array}{c} \left(\begin{array}{c} \frac{n+2}{2}\\ \frac{n-4}{2} \end{array}\right)\left(\begin{array}{c} \frac{n+2}{2}\\ \frac{n+2}{2} \end{array}\right)\left(\begin{array}{c} {\left(-{\tilde{P}}_{4}^{nd}\right)}^{\frac{n-4}{2}}{\left(-{\tilde{P}}_{5}^{nd}\right)}^{\frac{n+2}{2}}\\ +{\left(-{\tilde{P}}_{4}^{nd}\right)}^{\frac{n+2}{2}}{\left(-{\tilde{P}}_{5}^{nd}\right)}^{\frac{n-4}{2}} \end{array}\right)\\ +\left(\begin{array}{c} \frac{n+2}{2}\\ \frac{n-2}{2} \end{array}\right)\left(\begin{array}{c} \frac{n+2}{2}\\ \frac{n}{2} \end{array}\right)\left(\begin{array}{c} {\left(-{\tilde{P}}_{4}^{nd}\right)}^{\frac{n-2}{2}}{\left(-{\tilde{P}}_{5}^{nd}\right)}^{\frac{n}{2}}\\ +{\left(-{\tilde{P}}_{4}^{nd}\right)}^{\frac{n}{2}}{\left(-{\tilde{P}}_{5}^{nd}\right)}^{\frac{n-2}{2}} \end{array}\right) \end{array}\right) \end{array}]+\\ z^{2}[\begin{array}{c} -\left({\tilde{P}}_{1}^{d}+{\tilde{P}}_{2}^{d}\right)\left(\begin{array}{c} \frac{n+2}{2}\\ \frac{n}{2} \end{array}\right)\left(\begin{array}{c} \frac{n+2}{2}\\ \frac{n+2}{2} \end{array}\right)\left(\begin{array}{c} {\left(-{\tilde{P}}_{4}^{nd}\right)}^{\frac{n}{2}}{\left(-{\tilde{P}}_{5}^{nd}\right)}^{\frac{n+2}{2}}\\ +{\left(-{\tilde{P}}_{4}^{nd}\right)}^{\frac{n+2}{2}}{\left(-{\tilde{P}}_{5}^{nd}\right)}^{\frac{n}{2}} \end{array}\right)\\ +{\left(\begin{array}{c} \frac{n+2}{2}\\ \frac{n+2}{2} \end{array}\right)}^{2}{\left(-{\tilde{P}}_{4}^{nd}\right)}^{\frac{n+2}{2}}{\left(-{\tilde{P}}_{5}^{nd}\right)}^{\frac{n+2}{2}}\\ +{\tilde{P}}_{1}^{d}{\tilde{P}}_{2}^{d}\left(\begin{array}{c} \left(\begin{array}{c} \frac{n+2}{2}\\ \frac{n-2}{2} \end{array}\right)\left(\begin{array}{c} \frac{n+2}{2}\\ \frac{n+2}{2} \end{array}\right)\left(\begin{array}{c} {\left(-{\tilde{P}}_{4}^{nd}\right)}^{\frac{n-2}{2}}{\left(-{\tilde{P}}_{5}^{nd}\right)}^{\frac{n+2}{2}}\\ +{\left(-{\tilde{P}}_{4}^{nd}\right)}^{\frac{n+2}{2}}{\left(-{\tilde{P}}_{5}^{nd}\right)}^{\frac{n-2}{2}} \end{array}\right)\\ +{\left(\begin{array}{c} \frac{n+2}{2}\\ \frac{n}{2} \end{array}\right)}^{2}{\left(-{\tilde{P}}_{4}^{nd}\right)}^{\frac{n}{2}}{\left(-{\tilde{P}}_{5}^{nd}\right)}^{\frac{n}{2}} \end{array}\right) \end{array}]+\\ z^{1}[\begin{array}{c} {\tilde{P}}_{1}^{d}{\tilde{P}}_{2}^{d}\left(\begin{array}{c} \left(\begin{array}{c} \frac{n+2}{2}\\ \frac{n}{2} \end{array}\right)\left(\begin{array}{c} \frac{n+2}{2}\\ \frac{n+2}{2} \end{array}\right)\left(\begin{array}{c} {\left(-{\tilde{P}}_{4}^{nd}\right)}^{\frac{n}{2}}{\left(-{\tilde{P}}_{5}^{nd}\right)}^{\frac{n+2}{2}}\\ +{\left(-{\tilde{P}}_{4}^{nd}\right)}^{\frac{n+2}{2}}{\left(-{\tilde{P}}_{5}^{nd}\right)}^{\frac{n}{2}} \end{array}\right) \end{array}\right)\\ -\left({\tilde{P}}_{1}^{d}+{\tilde{P}}_{2}^{d}\right)\left({\left(\begin{array}{c} \frac{n+2}{2}\\ \frac{n+2}{2} \end{array}\right)}^{2}{\left(-{\tilde{P}}_{4}^{nd}\right)}^{\frac{n+2}{2}}{\left(-{\tilde{P}}_{5}^{nd}\right)}^{\frac{n+2}{2}}\right) \end{array}]+ \end{array} \end{array}$$

$$\begin{array}{c} \begin{array}{c} z^{0}[\left(\begin{array}{c} {\left(\begin{array}{c} \frac{n+2}{2}\\ \frac{n+2}{2} \end{array}\right)}^{2}{\tilde{P}}_{1}^{d}{\tilde{P}}_{2}^{d}\times \\ {\left(-{\tilde{P}}_{4}^{nd}\right)}^{\frac{n+2}{2}}{\left(-{\tilde{P}}_{5}^{nd}\right)}^{\frac{n+2}{2}} \end{array}\right)]=0. \end{array} \end{array}$$ (32)

Eq (32) can also be written as:

$$\begin{array}{c} \begin{array}{c} \Delta (z,m,\zeta_{cl},\omega_{cl},T_{s})={\tilde{A}}_{n+4}z^{n+4}+{\tilde{A}}_{n+3}z^{n+3}+\cdots +{\tilde{A}}_{2}z^{2}+{\tilde{A}}_{1}z^{1}+{\tilde{A}}_{0}z^{0}=0 \end{array}, \end{array}$$ (33)

where, ${\tilde{A}}_{j},j=\{(n+4),(n+2),\cdots ,2,1,0\}$ represent the coefficients of the characteristic Eq (33). Now, comparing the coefficients of (26) and (33) yields (30). *Theorem 5*: The dominant pole placement will be achieved for the given SOPTDZ system (1), if the filtered PID controller parameters {*K*_*p*_, *K*_*i*_, *K*_*d*_, *T*_*f*_} are obtained by solving the following nonlinear implicit equations when *m* is connected only with the real part of all complex conjugate non-dominant poles:

$$\begin{array}{c} \begin{array}{c} z^{0}:\;\psi_{1}=\tilde{K}{\tilde{K}}_{C}{\tilde{Z}}_{c1}{\tilde{Z}}_{c2}{\tilde{Z}}_{p1}+{\tilde{A}}_{0}=0,\\ z^{1}:\;\psi_{2}=\tilde{K}{\tilde{K}}_{C}({\tilde{Z}}_{c1}{\tilde{Z}}_{c2}+{\tilde{Z}}_{c1}{\tilde{Z}}_{p1}+{\tilde{Z}}_{c2}{\tilde{Z}}_{p1})-{\tilde{A}}_{1}=0,\\ z^{2}:\;\psi_{3}=\tilde{K}{\tilde{K}}_{C}({\tilde{Z}}_{c1}+{\tilde{Z}}_{c2}+{\tilde{Z}}_{p1})+{\tilde{A}}_{2}=0,\\ z^{3}:\;\psi_{4}=\tilde{K}{\tilde{K}}_{C}-{\tilde{A}}_{3}=0,\\ z^{n}:\;\psi_{5}={\tilde{P}}_{c1}{\tilde{P}}_{c2}{\tilde{P}}_{p1}{\tilde{P}}_{p2}-{\tilde{A}}_{n}=0,\\ z^{n+1}:\;\psi_{6}={\tilde{P}}_{p1}{\tilde{P}}_{p2}{\tilde{P}}_{c1}+{\tilde{P}}_{p1}{\tilde{P}}_{p2}{\tilde{P}}_{c2}+{\tilde{P}}_{c1}{\tilde{P}}_{c2}{\tilde{P}}_{p1}\\ \;\;\;\;+{\tilde{P}}_{c1}{\tilde{P}}_{c2}{\tilde{P}}_{p2}+{\tilde{A}}_{n+1}=0,\\ z^{n+2}:\;\psi_{7}={\tilde{P}}_{p1}{\tilde{P}}_{p2}+{\tilde{P}}_{p1}{\tilde{P}}_{c1}+{\tilde{P}}_{p1}{\tilde{P}}_{c2}+{\tilde{P}}_{p2}{\tilde{P}}_{c1}\\ \;\;\;\;+{\tilde{P}}_{p2}{\tilde{P}}_{c2}+{\tilde{P}}_{c1}{\tilde{P}}_{c2}-{\tilde{A}}_{n+2}=0,\\ z^{n+3}:\;\psi_{8}={\tilde{P}}_{c1}+{\tilde{P}}_{c2}+{\tilde{P}}_{p1}+{\tilde{P}}_{p2}+{\tilde{A}}_{n+3}=0. \end{array} \end{array}$$ (34)

*Proof*: Replace the complex conjugate non-dominant poles $\left\{{\tilde{P}}_{4}^{nd},{\tilde{P}}_{5}^{nd}\right\}$ by $\left\{{\tilde{P}}_{6}^{nd},{\tilde{P}}_{7}^{nd}\right\}$ in the desired characteristic polynomial (31). The proof is similar to that of Theorem 4.

*Remark 6*: During the random search and optimization procedure for simultaneously solving the polynomials, the binomial coefficient may not contain integer arguments in most real scenarios. To solve this numerical errors and efficient computing, the binomial coefficient factorials were represented in terms of the gamma functions as:

(nk)=Cnk=Γ(n+1)Γ(k+1)Γ(n−k+1),Γ(n)≜=∫0∞xn−1e−xdx,n∈ℝ+. (35)

The proposed algorithms are now validated by simulation and the results on a test bench SOPTDZ plants using three distinct PID controllers were derived from various non-dominant pole types detailed in the following section.

## 4 Simulations and results

### 4.1 The test-bench SOPTDZ plants

Twelve test-bench plants with various dynamical properties, such as stable, integrating, and unstable with a zero in the right or left half of the *s*-plane, have been taken into consideration to demonstrate the effectiveness of the proposed PID controller with a derivative filter. The parameters and characteristics of the test-bench SOPTDZ processes are detailed in Table 1. We have selected the sampling time as *T*_*s*_ = 0.02 sec, for designing the suggested discrete time filtered PID controller to handle all the test-bench plants. In order to get the time delay to sampling time ratio as an integer i.e. *n* = *L*/*T*_*s*_, $n\in Z_{+}$, the greatest common divisor (GCD) has been taken into account for the selection of *T*_*s*_ = 0.02 sec here. This ensures that all the test-bench plants with various delays, given in Table 1 are divided by a common factor of 0.02.

**Table 1:** Characteristics and parameters of the test-bench SOPTDZ plants.

Process Models	*K*	*τ*	*L*	*T*	*ζ*_*ol*_	*L*/*T*	|*T*/*τ*|	Open-Loop Characteristics
$G_{1}=\frac{\left(2s+1\right)e^{-s}}{\left(10s+1)(0.5s+1\right)}$ [52]	1	2	1	2.2361	2.3479	0.4472	> 1	Stable, Over-damped, Minimum phase
$G_{2}=\frac{\left(-s+1\right)e^{-s}}{10s^{2}+7s+1}$ [53]	1	−1	1	3.1623	1.1068	0.3162	> 1	Stable, Over-damped, Non-minimum phase
$G_{3}=\frac{\left(-4s+1\right)e^{-s}}{9s^{2}+2.4s+1}$ [54]	1	−4	1	3	0.4	0.3333	< 1	Stable, Under-damped, Non-minimum phase
$G_{4}=\frac{\left(-0.2s+1\right)e^{-0.2s}}{\left(s+1)(s+1\right)}$ [55]	1	−0.2	0.2	1	1	0.2	> 1	Stable, Critically-damped, Non-minimum phase
$G_{5}=\frac{\left(-s+1\right)e^{-3s}}{s(2s+1)}$ [53]	10^12^	−1	3	1.4142×10^6^	3.5355×10^5^	2.1213×10^−6^	> 1	Integrating stable, Over-damped, Non-minimum phase
$G_{6}=\frac{0.25(s+1)}{s(2s+1)}$ [54]	0.25×10^12^	−1	0	1.4142×10^6^	3.5355×10^5^	0	> 1	Integrating stable, Over-damped, Minimum phase
$G_{7}=\frac{-1.6(-0.5s+1)}{s(3s+1)}$ [52]	−1.6×10^12^	−0.5	0	1.7321×10^6^	2.8868×10^5^	0	> 1	Integrating stable, Over-damped, Non-minimum phase
$G_{8}=\frac{\left(10s+1\right)e^{-s}}{s(2s+1)}$ [52]	10^12^	10	1	1.4142×10^6^	3.5355×10^5^	7.0711×10^−7^	> 1	Integrating stable, Over-damped, Minimum phase
$G_{9}=\frac{6.83\times 10^{-4}\left(766.0752s+1\right)e^{-s}}{s(1112.099s-1)}$ [56]	6.83×10^8^	766.0752	1	3.3348×10^7^	−1.4993×10^4^	2.9987×10^−8^	> 1	Integrating unstable, Minimum phase
$G_{10}=\frac{2\left(5s+1\right)e^{-0.3s}}{\left(3s-1)(s-1\right)}$ [57]	2	5	0.3	1.7321	−1.1547	0.1732	< 1	Double unstable, Minimum phase
$G_{11}=\frac{0.03\left(s+1\right)e^{-0.4s}}{0.516s^{2}-0.0945s+1}$ [57]	0.03	1	0.4	0.7183	−0.0658	0.5569	<1	Unstable, Minimum phase
$G_{12}=\frac{2.07\left(0.1507s+1\right)e^{-0.3s}}{-2.85s^{2}-2.31s+1}$ [58]	2.07	0.1507	0.3	1.6882*j*	0.684*j*	−0.177*j*	> 1	Unstable, Minimum phase

### 4.2 Control loop performance measures

**Fig 1:**
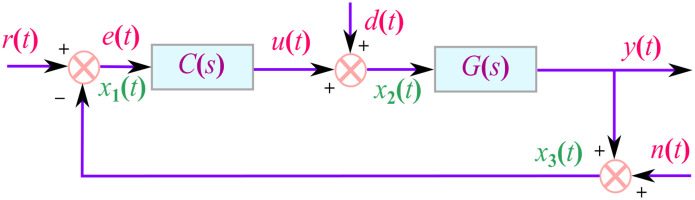
Closed loop structure of SOPTD with lead plant with filtered PID controller.

In addition to stability study, utilizing the three distinct filtered PID controllers with different non-dominant pole types, analysis of various control loop performance metrics is also a key component in the design of control systems [23–25, 46, 47]. As illustrated in Fig 1, the closed loop systems with three separate external inputs, namely set-point, disturbance, and noise inputs, play a critical role in maintaining the internal stability. Nine transfer functions are known to fulfill this role and different performance measures can be calculated from these transfer functions as:

$$\begin{array}{c} {}[\begin{array}{c} x_{1}\\ x_{2}\\ x_{3} \end{array}]=[\begin{array}{c} e\\ u+d\\ y+n \end{array}]=\frac{1}{1+GC}[\begin{array}{ccc} 1 & -G & -1\\ C & 1 & -C\\ GC & G & 1 \end{array}][\begin{array}{c} r\\ d\\ n \end{array}]. \end{array}$$ (36)

Only four of the nine transfer functions have the most influence on how different control loop performance metrics are shaped by balancing their trade-offs [23–25, 46, 47] and these are known as the sensitivity *S*_*e*_(*s*), complementary sensitivity *T*(*s*), control sensitivity *S*_*u*_(*s*) and disturbance sensitivity *S*_*d*_(*s*) functions, as follows:

$$\begin{array}{c} \begin{array}{c} S_{e}(s)=1/(1+G_{ol}(s))=G_{re}=G_{dx_{2}}=G_{nx_{3}},\\ T(s)=G_{ol}(s)/(1+G_{ol}(s))=G_{rx_{3}},\;\\ S_{e}(s)+T(s)=1,\\ S_{u}(s)=C(s)/(1+G_{ol}(s)),\;\\ S_{d}(s)=G(s)/(1+G_{ol}(s)), \end{array} \end{array}$$ (37)

where, *G*_*ol*_(*s*) = *C*(*s*)*G*(*s*) is the open loop system.

As a result, it is possible to compute the responses of the manipulated variable *u*(*t*) and the controlled variable *y*(*t*) in response to a unit change in the set-point *r*(*t*) and disturbance inputs *d*(*t*) as:

$$\begin{array}{c} \begin{array}{c} y_{r}(t)=L^{-1}(R(s)T(s)),y_{d}(t)=L^{-1}(D(s)S_{d}(s)),\\ u_{r}(t)=L^{-1}(R(s)S_{u}(s)),u_{d}(t)=L^{-1}(D(s)S_{e}(s)), \end{array} \end{array}$$ (38)

where, $L^{-1}(\cdot )$ is the inverse Laplace transform operator, and {R(s)=L(r(t))=1/s, D(s)=L(d(t))=1/s} represent the Heaviside step function in the reference and disturbance inputs respectively.

### 4.3 Choice of objective function and selecting the best compromise filtered PID controller parameters

The derived polynomials (23), (30) and (34), obtained from three different non-dominant pole types cannot be explicitly used to obtain the filtered PID controller parameters {*K*_*p*_, *K*_*i*_, *K*_*d*_, *T*_*f*_} because these polynomials are implicit in nature and need to be solved simultaneously for a chosen closed loop design specifications {*m*, *ζ*_*cl*_, *ω*_*cl*_} and sampling time (*T*_*s*_). Also, it may not always be possible to obtain feasible solutions from (23), (30) and (34) for arbitrary closed loop specifications. As a result, the closed loop design specifications {*m*, *ζ*_*cl*_, *ω*_*cl*_} can vary widely [48, 49] when choosing using a randomized search algorithm. By minimizing the set-point tracking performance measurements, namely the integral of squared error (ISE) as also used in [24, 25], we use the particle swarm optimization (PSO) based random search approach to achieve optimal values of both the design and controller parameters i.e. *θ*_*opt*_ = {*m*, *ζ*_*cl*_, *ω*_*cl*_, *K*_*p*_, *K*_*i*_, *K*_*d*_, *T*_*f*_} simultaneously. However, a controller tuned with the goal of the minimal ISE criterion will not always meet the polynomial criteria and vice versa. Thus, in the hybrid performance index below, as opposed to [24, 25], we give greater importance on the dominant pole placement component (Ψ) as compared to the ISE criteria for determining the optimum compromise solution within a range of controller gains vs. fulfillment of the chosen polynomial criteria (Ψ), i.e.

$$\begin{array}{c} \begin{array}{c} \theta_{opt}=\underset{\theta \in \Theta }{\text{arg}\;\text{min}}\;J(\theta ),\;\;\\ J(\theta )=w\int_{0}^{T}e^{2}(\theta ,t)dt+(1-w)\Psi (\theta ),\\ T=100,w=0.1,\Psi (\theta )=\sum_{i=1}^{8}\psi_{i}^{2}(\theta ). \end{array} \end{array}$$ (39)

Similar numerical strategies were previously investigated in [50, 51], where different performance criteria were taken into account as weighted averages to construct a hybrid objective function for optimization based controller tuning. In order to maintain the closed loop stability and prevent the set-point perturbation responses from being unbounded, the objective function (39) considers the ISE performance measure. Otherwise, a heavy penalty of ISE = 10^6^ is applied, and the random search is redirected towards the stabilizable regions. The randomized guess controller parameters’ ability to fulfill a set of simultaneous nonlinear implicit equations produced from the dominant pole placement criteria is controlled by the second part (Ψ) of Eq (39). From all the sampled stable data-points which were visited by the PSO algorithm in the combined high-dimensional space of controller settings and design requirements, a trade-off may be obtained between the two components of the objective function as discussed in the next section. Special attention should be given while choosing the best compromise solution by varying *w* from these sampled points for *w* = {0.1, 0.5, 0.9} within the range of design and PID controller parameters Θ = {*m* ∈ [0, 10], *ζ*_*cl*_ ∈ [0, 10], *ω*_*cl*_ ∈ [0, 10], *K*_*p*_ ∈ [−10, 10], *K*_*i*_ ∈ [−10, 10], *K*_*d*_ ∈ [−10, 10], *T*_*f*_ ∈ [0, 10]}. This illustrates the circumstances in which the ISE is less important or more important than meeting the summed algebraic formulas for dominant pole placement Ψ. Here, the aim is to create an objective function with two components which provide different degree of emphasis on the two components between control performance and conditions of achieving the dominant pole placement by satisfying the polynomials. Therefore, it is ensuring the minimum ISE criterion as well as satisfying the analytical pole placement conditions. We fix *w* = 0.1 in Eq (39) during the optimization process, so that the dominant pole placement criteria is emphasized over minimizing the ISE criteria for unit set-point change to enable post-hoc filtering of the obtained solutions using other closed loop performance metrics. Detail description of the parameters for the optimization design are reported in [24, 25, 38]. However, analyzing the computational complexity of the proposed controller design approach is beyond the scope of this paper.

Moreover, the PSO is an efficient multi-agent search and optimization method and easy to implement. The effect of different optimizer for the same computational problem is beyond the scope of the current research. However, with decent number of particles, depending on the dimension of the search space, the obtained stability regions should not change too much, irrespective of the chosen optimization algorithm.

### 4.4 Time domain and frequency domain responses

In this subsection, the proposed three different filtered PID controller obtained from three distinct non-dominant pole types have been used to evaluate the control loop performances for all the twelve SOPTDZ test-bench plants shown in Table 1. The dynamical characteristics and parameters of twelve test-bench SOPTDZ processes are depicted in Table 1 which include stable, integrating, unstable with minimum as well as non-minimum phase zeros. Moreover, these test-bench processes are classified with different filter characteristic such as low-pass, band-pass and band-stop for different ratios between the lag, delay, lead and damping. System with zero in the right half of the *s*-plane can be defined as non-minimum phase system and zero on the left half of the *s*-plane is defined as minimum phase system. These are a special class of linear time-invariant (LTI) systems, which have several industrial applications e.g. DC–DC boost converters, water level in drum boiler, valve control system, telescope azimuth angle control system, and some biological and chemical processes [53]. For example, in a drum boiler, the overall liquid level and the volume of water at boiling unit will be decreased for a short span of time period if the flow rate of cold water is increased. The drop in temperature is the cause of this since it affects the volume of concentrated water needed for the conversion of vapours. The heat supply and production of steam remain constant during this phenomenon. Hence, the liquid level at the boiling unit will begin to increase. Therefore, the combination of these two opposing processes results in the non-minimum phase characteristics and it is quite challenging to handle such systems. Here, we have considered the twelve test-bench SOPTDZ processes and all the test-bench plants (*G*_1_ − *G*_12_) represent the dynamical model of real world industrial processes.

**Fig 2:**
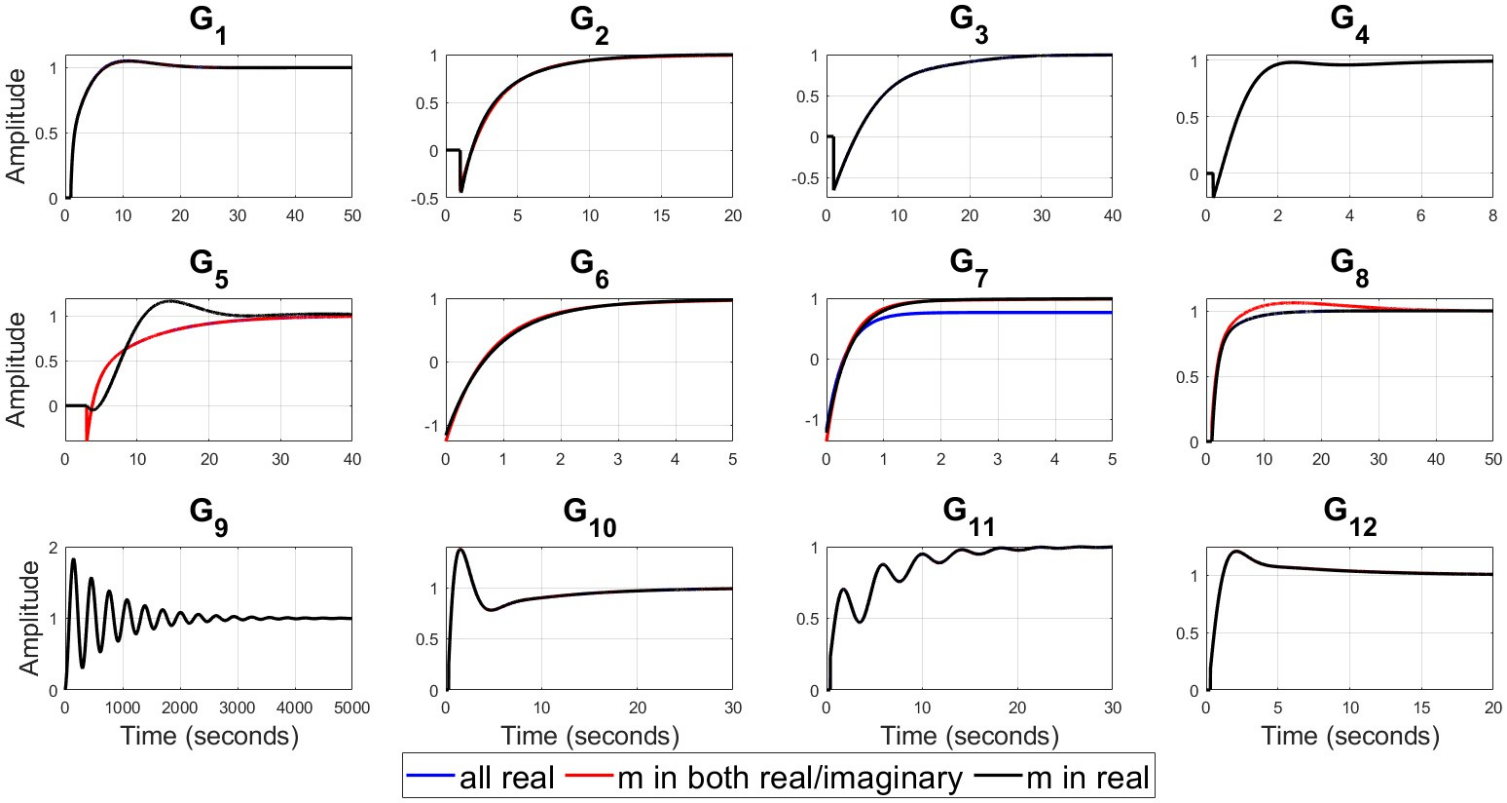
Controlled output *y*(*t*) for the 12 test-bench plants with step change in reference input.

**Fig 3:**
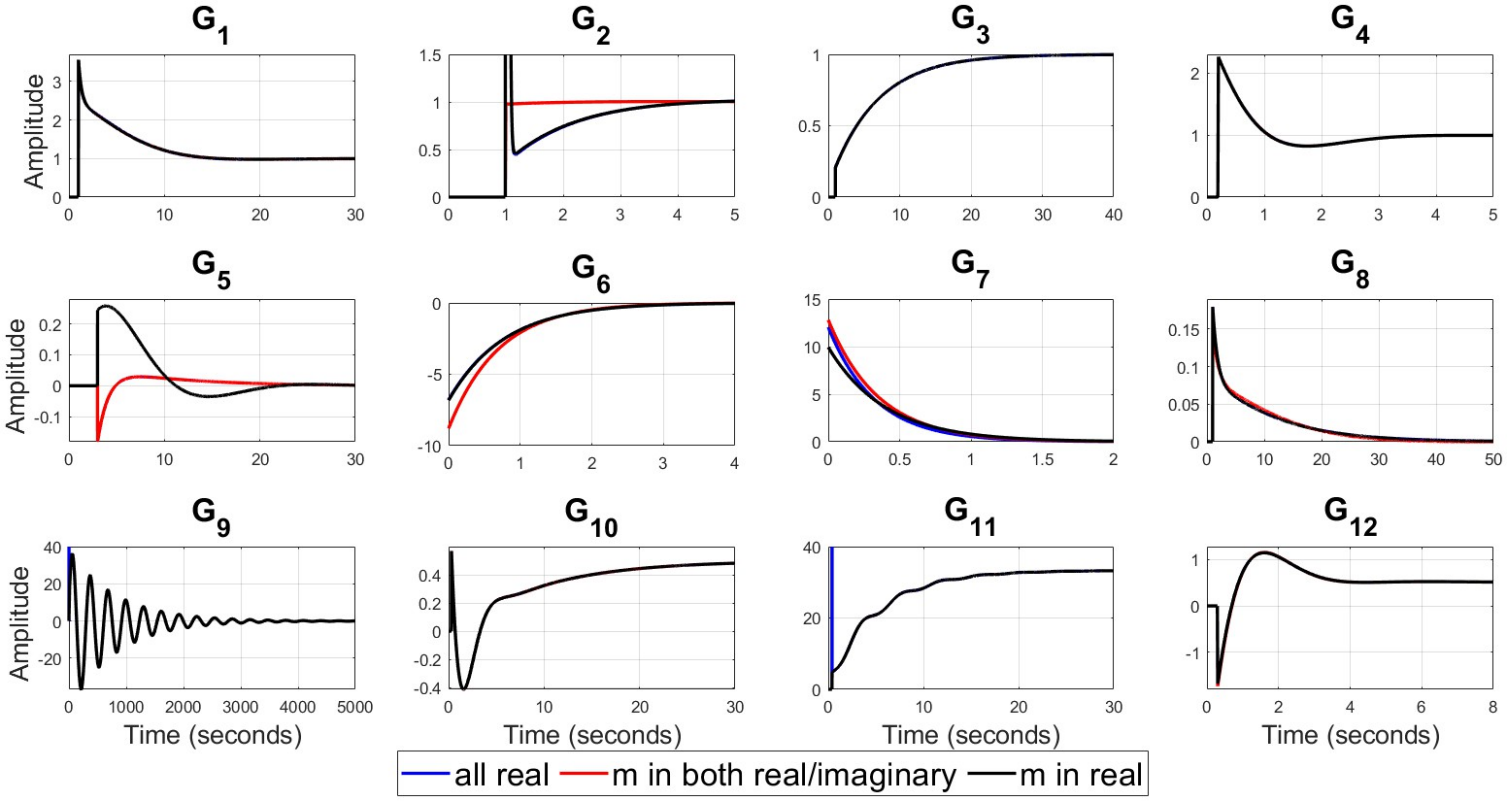
Manipulated output *u*(*t*) for the 12 test-bench plants with step change in reference input.

**Fig 4:**
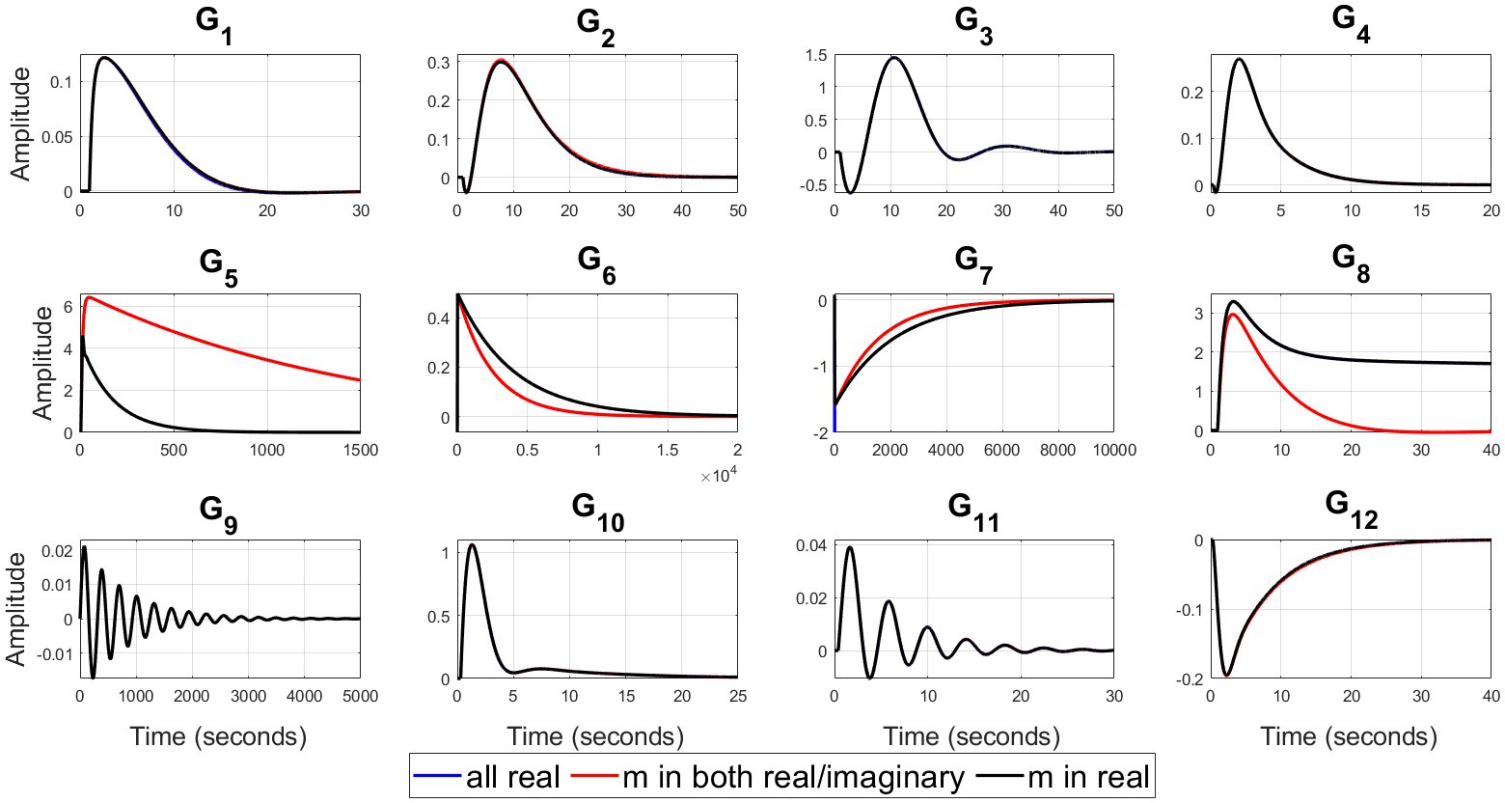
Controlled output *y*(*t*) for the 12 test-bench plants with step change in disturbance input.

**Fig 5:**
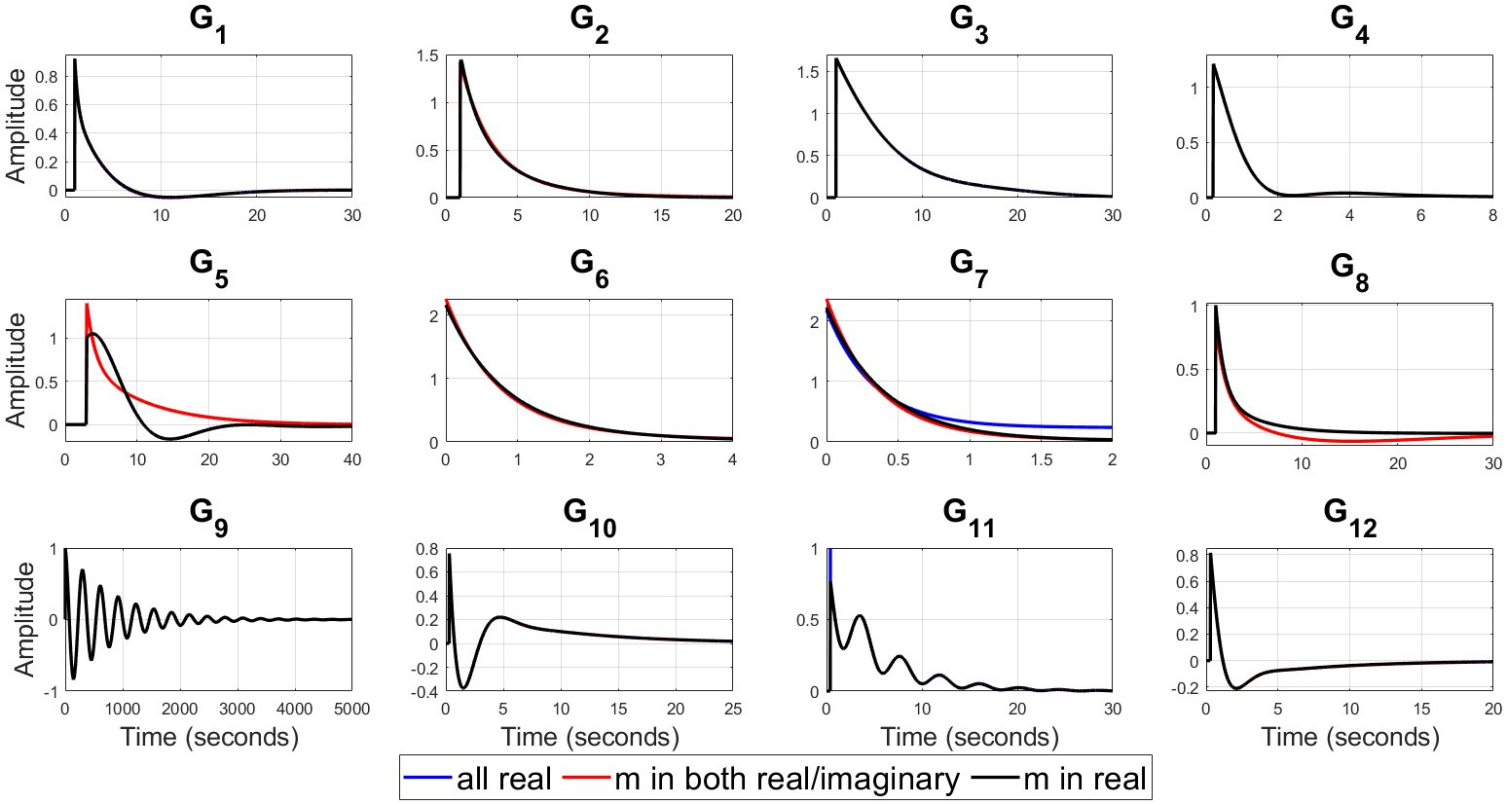
Manipulated output *u*(*t*) for the 12 test-bench plants with step change in disturbance input.

**Fig 6:**
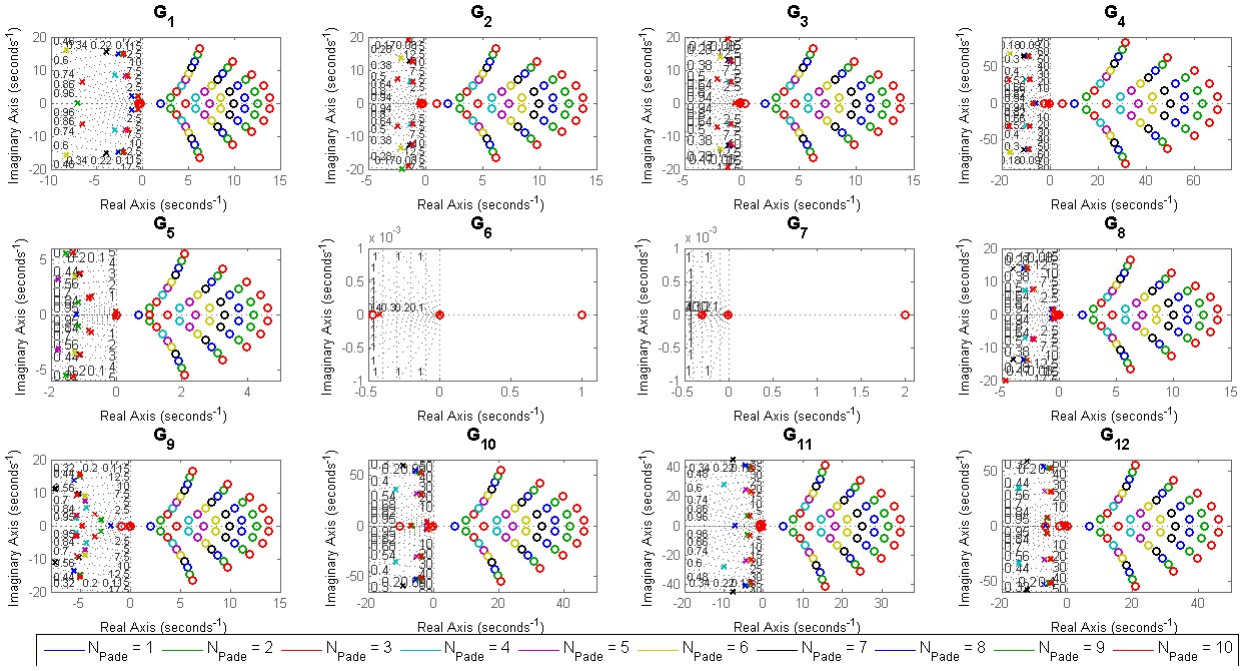
Pole-zero map for the 12 test-bench plants with filtered PID controller obtained by all complex conjugate pole type with *m* in real part.

**Fig 7:**
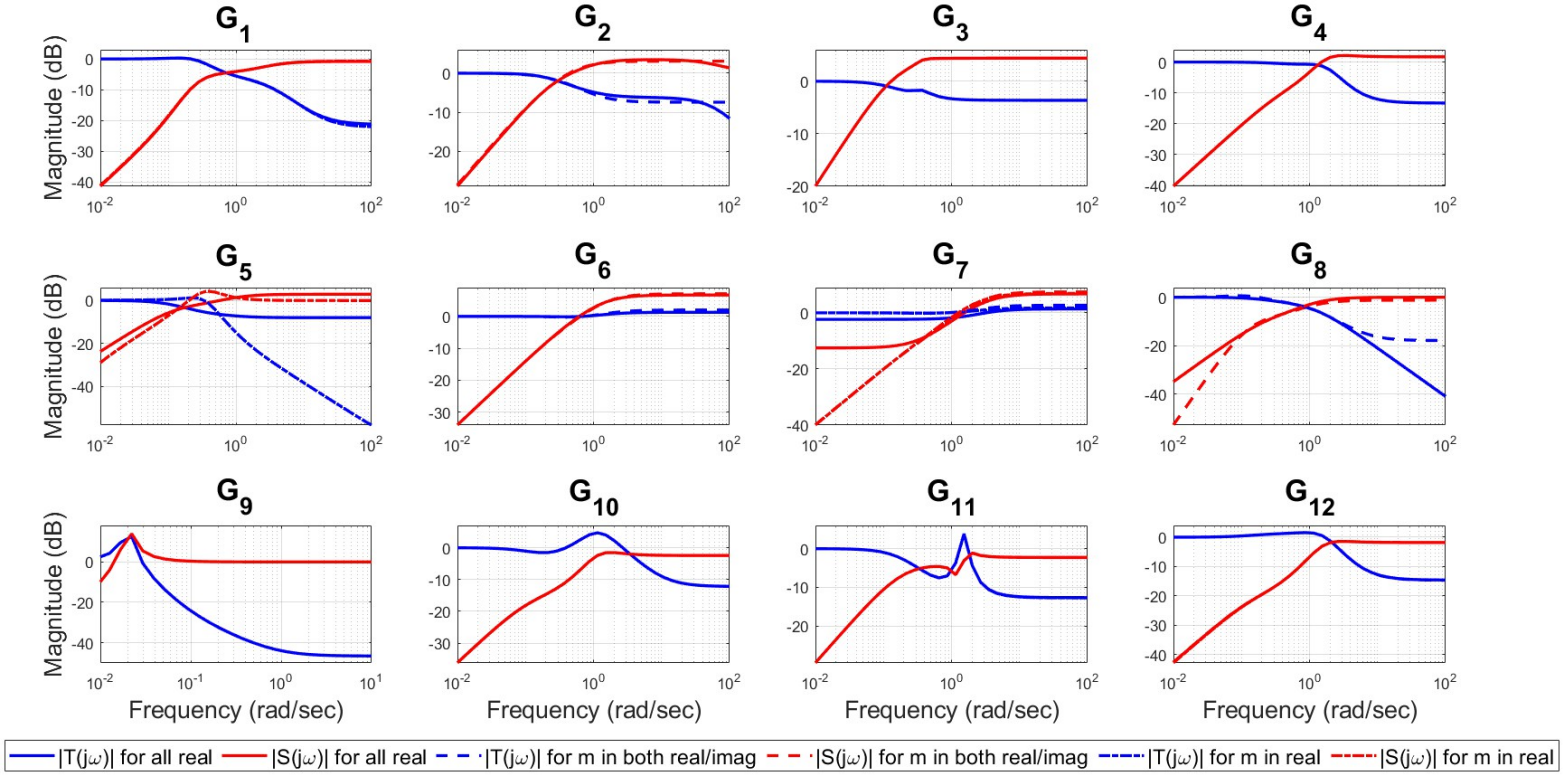
Sensitivity and complementary sensitivity trade-offs for the 12 test-bench plants.

For example, (*G*_3_, *G*_6_) represent a valve control system and water level in boiler drum respectively [54], *G*_9_ is representing the dynamics of jacketed continuous stirred tank reactor (CSTR) [56], (*G*_10_ − *G*_12_) represent isothermal CSTR [57, 58] etc. The optimal values of the closed loop design specifications {*m*, *ζ*_*cl*_, *ω*_*cl*_}, filtered PID controller gains {*K*_*p*_, *K*_*i*_, *K*_*d*_, *T*_*f*_}, ISE, and Ψ for all of twelve test-bench plants are obtained by applying the proposed methods, as shown in Table 2. It can be observed from Table 2 that *ζ*_*cl*_ for all real non-dominant pole type is mostly over-damped, for all complex non-dominant pole type where *m* in both (real and complex) and *m* in real are mostly under-damped. Also, it is observed that the filter time constant *T*_*f*_ is zero for most of the SOPTDZ processes for set-point response which means that these SOPTDZ processes are self-stabilizing even without the derivative filter. Though, the value of *T*_*f*_ may differ for similar stabilization method using the load disturbance response. For all test-bench plants utilizing three different filtered PID controllers, the controlled variable *y*(*t*) and manipulated variable *u*(*t*) due to step change in the reference input *r*(*t*) have been shown in Figs 2 and 3 respectively. Figs 4 and 5 show the time response of the controlled variable *y*(*t*) and manipulated variable *u*(*t*) under step changes in the disturbance input *d*(*t*) respectively. We used the *iodelay* command from MATLAB’s control system toolbox in order to represent exponential term of 12 test-bench SOPTDZ processes and the corresponding time response may not be identical under different Pade approximation for such complex plants. With the exception of the delay-free plants (*G*_6_ − *G*_7_), it is seen from Figs 2–5 that the filtered PID controller obtained from the complex-conjugate non-dominant pole type where *m* is only connected with the real part performs better in terms of tracking and disturbance rejection than the other two classes of PID controllers. Whereas, the filtered PID controller, obtained from the complex-conjugate non-dominant pole type where *m* is connected in both the real and imaginary part provides better tracking and disturbance rejection performance for delay free systems than other two class of controller. It is noted that, the integrating plant *G*_7_ controlled by the filtered PID controller designed from all real non-dominant pole type does not ensure the tracking performance since the obtained integral gain *K*_*i*_ is zero whereas the PID controllers obtained from all complex-conjugate non-dominant pole types are able to ensure good tracking performance. The closed loop pole locations of all 12 test-bench SOPTDZ plants are shown in Fig 6. These closed loop pole locations for all test bench plants are obtained by varying the Pade approximation order for the delay term i.e. *N*_*Pade*_ = 1 to *N*_*Pade*_ = 10 when all the plants are controlled by a filtered PID controller that is designed with all complex-conjugate non-dominant pole types with *m* only present in the real part. Using (37), Fig 7 shows the trade-off between magnitude plots of the sensitivity |*S*_*e*_(*jω*)| and complementary sensitivity |*T*(*jω*)| functions [23–25]. The high-pass characteristics of the sensitivity and the low-pass characteristics of the complementary sensitivity are related to their ability to reject noise and disturbances, respectively. The filtered PID controller, designed with the all complex-conjugate non-dominant pole type with (*m*) in the real part only, has been found to be superior to the other two types of controller, similar to the time responses. This is especially true for the most difficult plants to control, i.e., open loop unstable plants (*G*_9_ − *G*_12_). Here, the purpose is to show robust stabilization of SOPTDZ systems with filtered PID controller. Thus, other metrics, such as the impact of measurement noise, have not been investigated because they are not the primary focus of this study.

**Table 2:** Optimum design specifications and filtered PID controller parameters along with the two components of the objective function with the weight *w* = 0.1 and different non-dominant pole types for the SOPTDZ test-bench processes.

Non-dominant Pole Types	Plant	ISE	Ψ	*m*	*ζ*_*cl*_	*ω*_*cl*_	*K*_*p*_	*K*_*i*_	*K*_*d*_	*T*_*f*_
All Real	*G*_1_	63.6382	1.0746	9.9994	4.2116	4.6647	4.1342	1.1668	0.2372	0.0000
	*G*_2_	166.6818	2.8432	9.9998	9.5573	2.0054	1.9937	0.2773	3.2539	0.0104
	*G*_3_	470.4823	4.0923	10.0000	10.0000	1.9582	0.2587	0.0997	0.8927	0.0000
	*G*_4_	38.4238	1.2619	10.0000	9.0201	1.4059	2.6277	1.0412	0.8861	0.0000
	*G*_5_	284.3550	2.6766	10.0000	9.3793	2.6324	0.1527	0.0001	0.5714	0.0000
	*G*_6_	100.3871	5.7406	2.1680	1.6432	10.0000	2.0003	0.0005	4.2936	0.0000
	*G*_7_	41.2929	5.8042	2.1674	1.6365	10.0000	0.0000	0.0000	-2.0243	0.0000
	*G*_8_	72.8556	9.6596	10.0000	3.8602	4.5637	0.5412	0.0009	-3.6149	9.9999
	*G*_9_	1671.413	0.9781	9.9998	8.1092	2.4124	10.0000	0.8728	10.0000	0.0007
	*G*_10_	64.0714	3.0468	10.0000	7.4482	1.9037	1.0499	0.3196	0.0977	0.0000
	*G*_11_	71.3520	2.6918	9.9980	4.6303	3.3381	9.9978	9.9998	5.2049	0.0001
	*G*_12_	28.5188	1.8079	10.0000	6.7110	2.1281	-5.3593	-0.6475	-2.0953	0.0000
All Complex (*m* in Both Real & Complex Conjugate)	*G*_1_	63.7246	11.0379	10.0000	0.5689	10.0000	4.1640	1.1383	0.2203	0.0000
	*G*_2_	168.6861	12.8422	10.0000	0.5694	10.0000	1.9975	0.2664	2.9874	0.0000
	*G*_3_	470.4850	14.2752	10.0000	0.5696	10.0000	0.2580	0.0997	0.8926	0.0000
	*G*_4_	38.4234	2.1199	10.0000	0.7632	10.0000	2.6275	1.0386	0.8861	0.0000
	*G*_5_	284.3661	380.601	10.0000	0.5977	10.0000	0.1527	0.0001	0.5712	0.0000
	*G*_6_	100.9204	4.8264	6.8501	0.2289	10.0000	1.9926	0.0008	4.4542	0.0000
	*G*_7_	51.1108	4.8009	6.8280	0.2198	10.0000	-0.6210	-0.0004	-2.1637	0.0000
	*G*_8_	66.2828	11.5397	10.0000	0.5695	10.0000	0.2011	0.0428	0.0293	0.0000
	*G*_9_	1671.4175	11.1897	10.0000	0.5697	10.0000	10.0000	0.8728	10.0000	0.0000
	*G*_10_	64.1489	5.2191	10.0000	0.6903	10.0000	1.0491	0.3210	0.0975	0.0000
	*G*_11_	72.2073	6.2737	10.0000	0.6449	10.0000	10.0000	10.0000	5.1508	0.0000
	*G*_12_	29.0162	3.9316	10.0000	0.6859	10.0000	-5.3591	-0.6417	-2.0775	0.0000
All Complex (*m* in Real)	*G*_1_	63.9218	1.0349	10.0000	9.3204	2.1474	4.1614	1.1348	0.2121	0.0000
	*G*_2_	166.7344	2.8420	10.0000	5.1632	3.7732	1.9931	0.2766	3.2480	0.0104
	*G*_3_	470.4850	4.0930	10.0000	10.0000	1.9929	0.2580	0.0997	0.8926	0.0000
	*G*_4_	38.4219	1.2588	9.9998	6.9225	1.8515	2.6278	1.0359	0.8862	0.0000
	*G*_5_	916.7127	11.1847	9.9381	6.1078	6.5646	0.2446	0.0014	0.0007	0.0000
	*G*_6_	100.3967	5.7706	3.0402	1.3060	10.0000	2.0002	0.0005	4.2973	0.0000
	*G*_7_	50.4719	5.8024	3.0387	1.2892	10.0000	-0.6270	-0.0003	-2.0610	0.0000
	*G*_8_	72.8566	9.6562	10.0000	3.3321	5.3645	0.5400	0.0009	-3.6051	9.9999
	*G*_9_	1671.4128	0.9784	10.0000	10.0000	1.9917	10.0000	0.8728	10.0000	0.0000
	*G*_10_	64.0573	3.0442	10.0000	9.2985	1.5442	1.0500	0.3214	0.0978	0.0000
	*G*_11_	72.2033	2.6481	10.0000	9.7507	1.6081	9.9999	10.0000	5.1515	0.0000
	*G*_12_	28.4955	1.8068	10.0000	8.6358	1.6748	-5.3470	-0.6612	-2.0980	0.0000

### 4.5 Stabilizing regions for the test bench SOPTDZ processes

**Fig 8:**
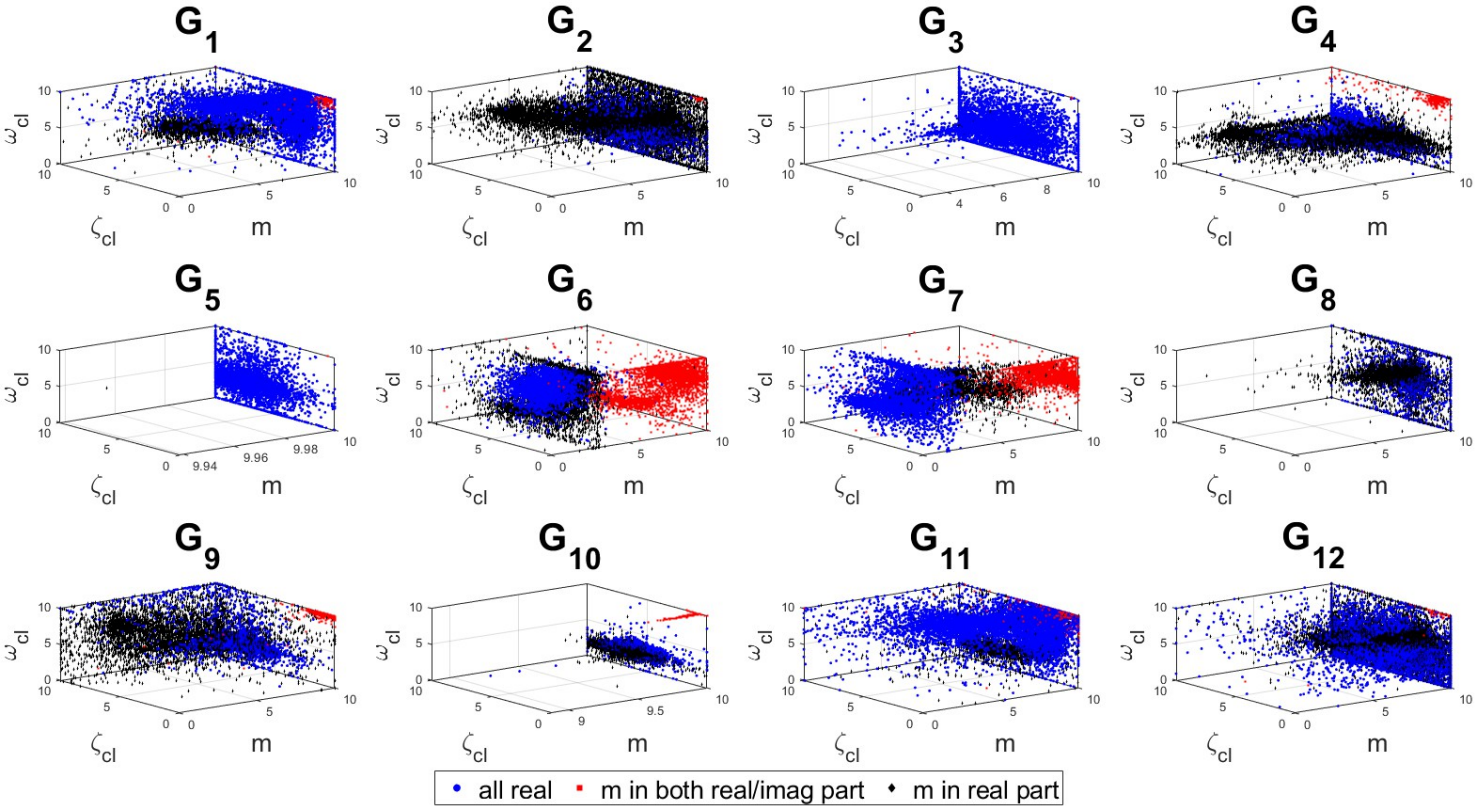
Sampled points in the design parameter space with a threshold ISE< 10^4^.

**Fig 9:**
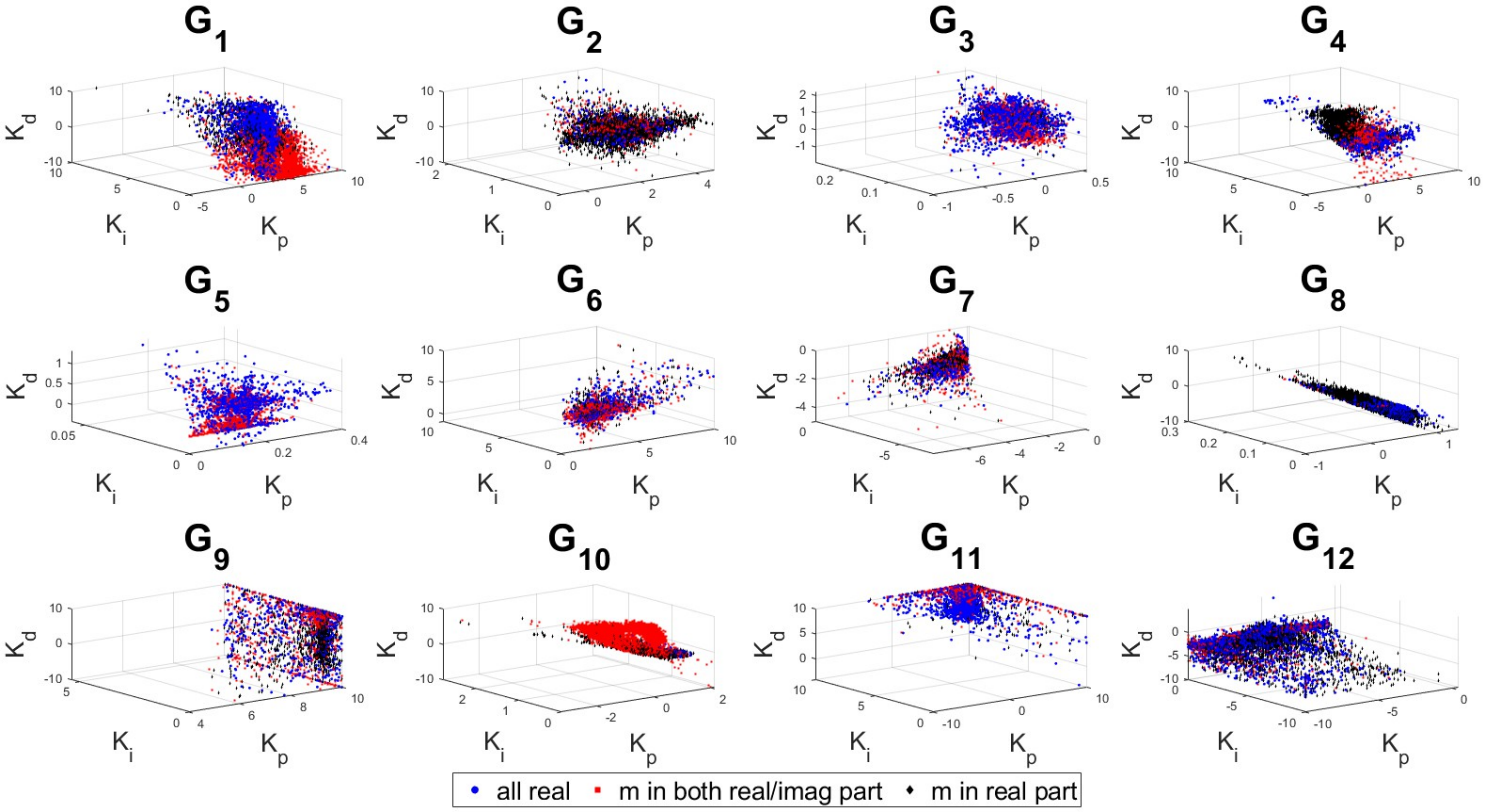
Sampled points in the controller parameter space with a threshold ISE< 10^4^ for combination 1.

**Fig 10:**
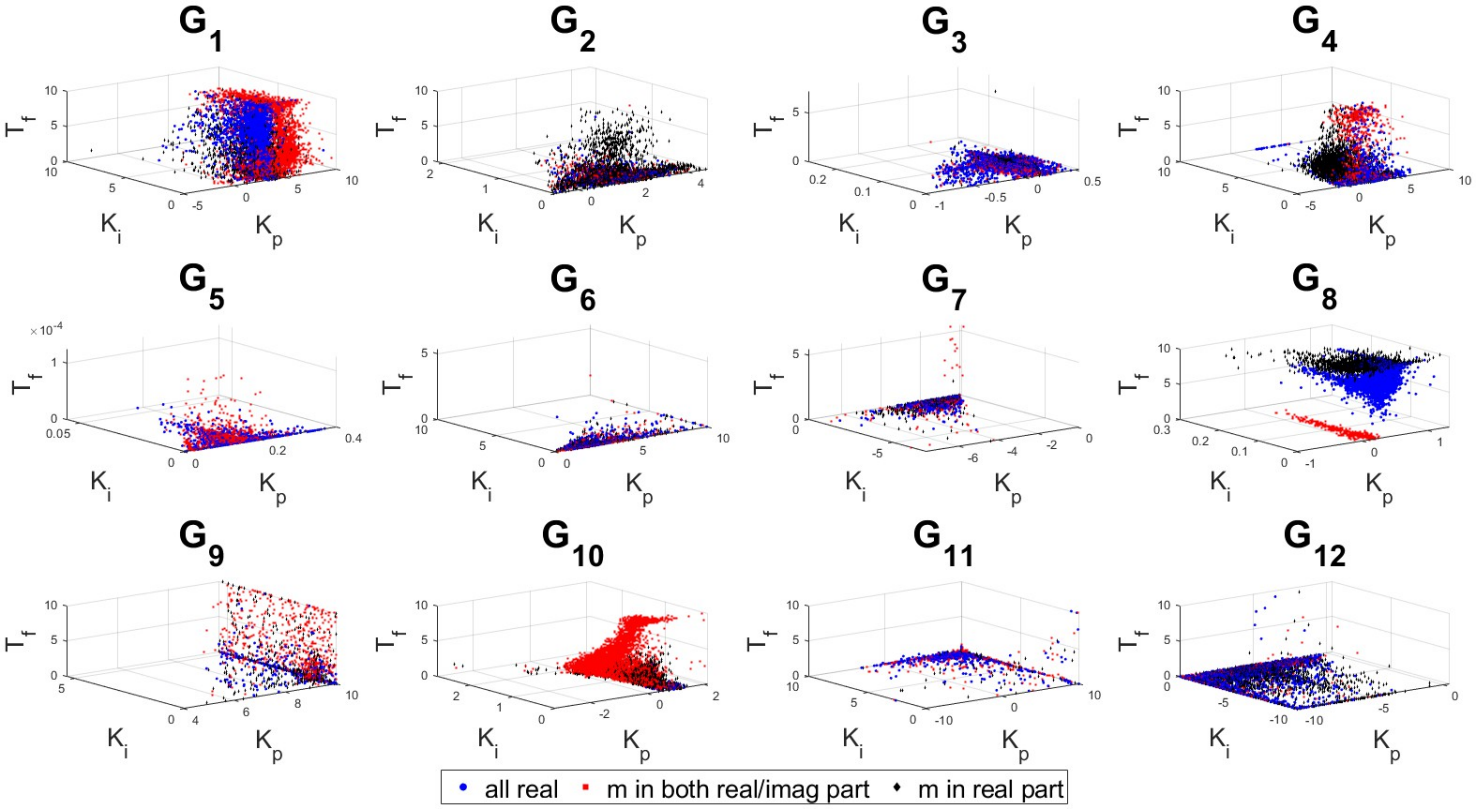
Sampled points in the controller parameter with a threshold ISE< 10^4^ for combination 2.

**Fig 11:**
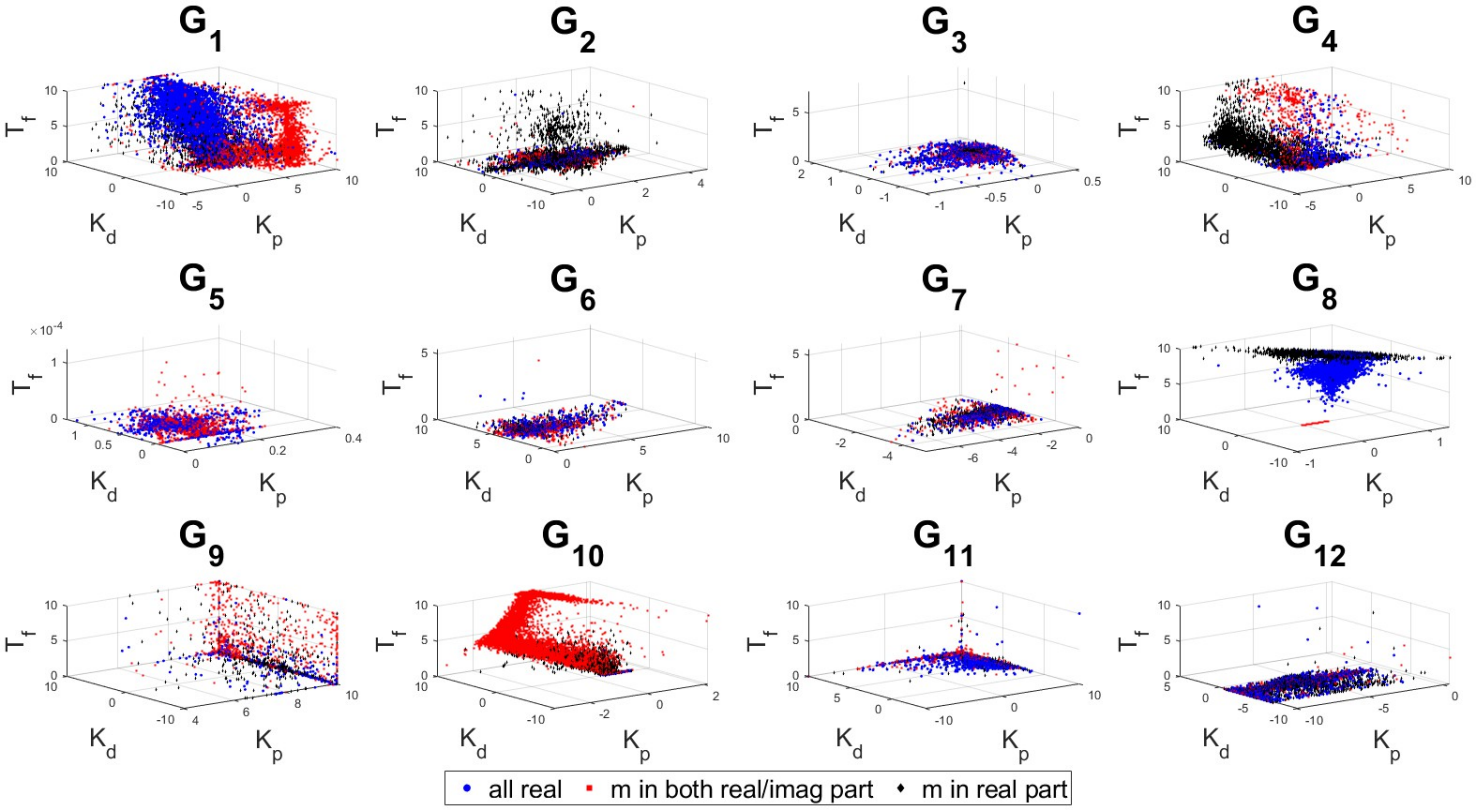
Sampled points in the controller parameter with a threshold ISE< 10^4^ for combination 3.

**Fig 12:**
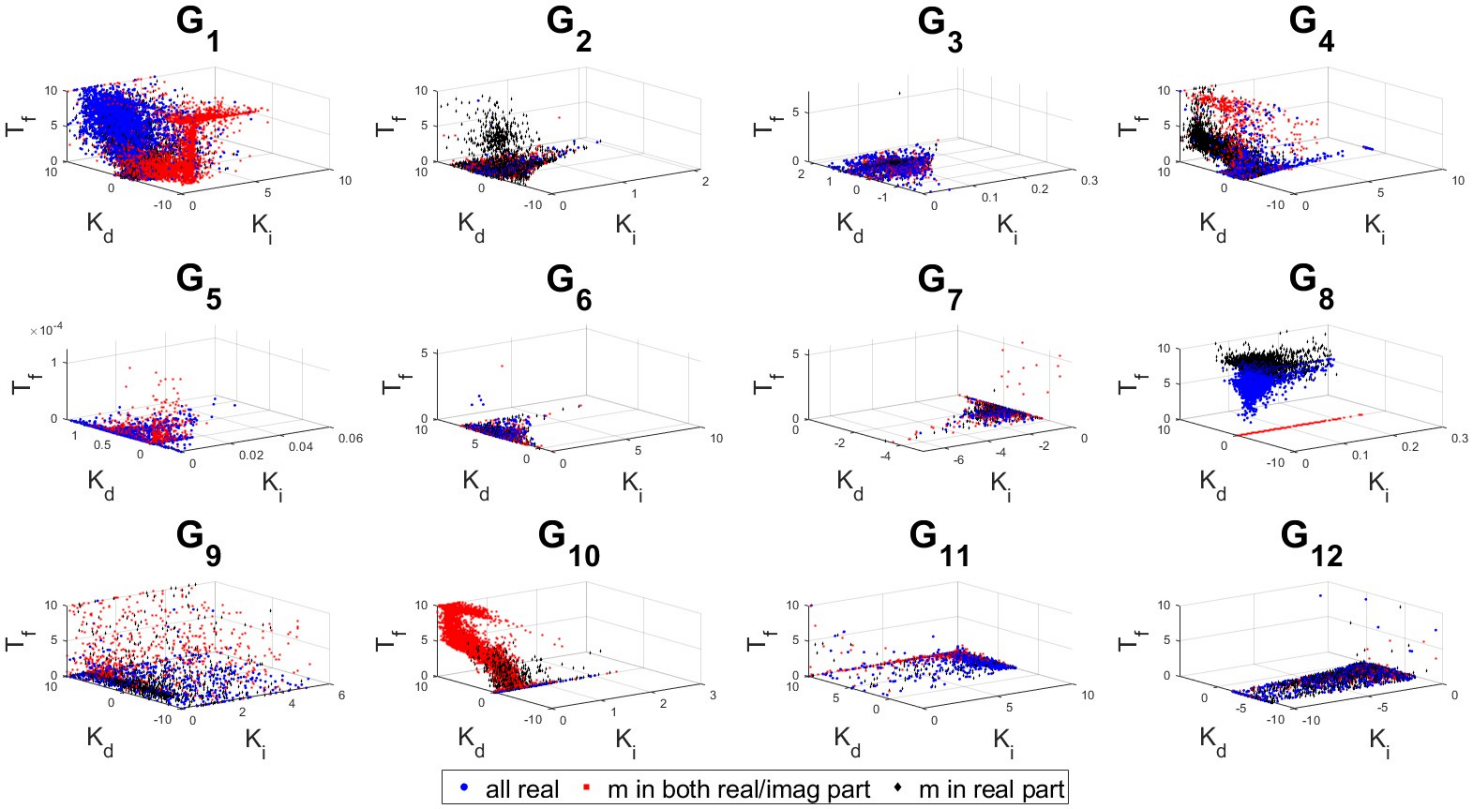
Sampled points in the controller parameter space with a threshold ISE< 10^4^ for combination 4.

The 3D stability regions for all the test-bench plants in design and controller parameter space are shown in Figs 8–12 respectively, in different combination of the controller parameters. Here, we apply the threshold of ISE < 10^4^ on the obtained samples explored by the PSO based random search process, using the hybrid objective function (39). The obtained stability region is approximated by random sampling approach. This represents a significant advancement over earlier works [24–26]. Considering the delayed systems without lead and PID controller without a low-pass filter, the 3D stability regions were obtained in the design specification and PID controller parameter space in [24, 25]. In [26], the 2D stability regions were obtained in proportional, derivative and filter parameter space with eigen-value assignment.

### 4.6 Design trade-off between control performance and satisfying the pole placement conditions

**Fig 13:**
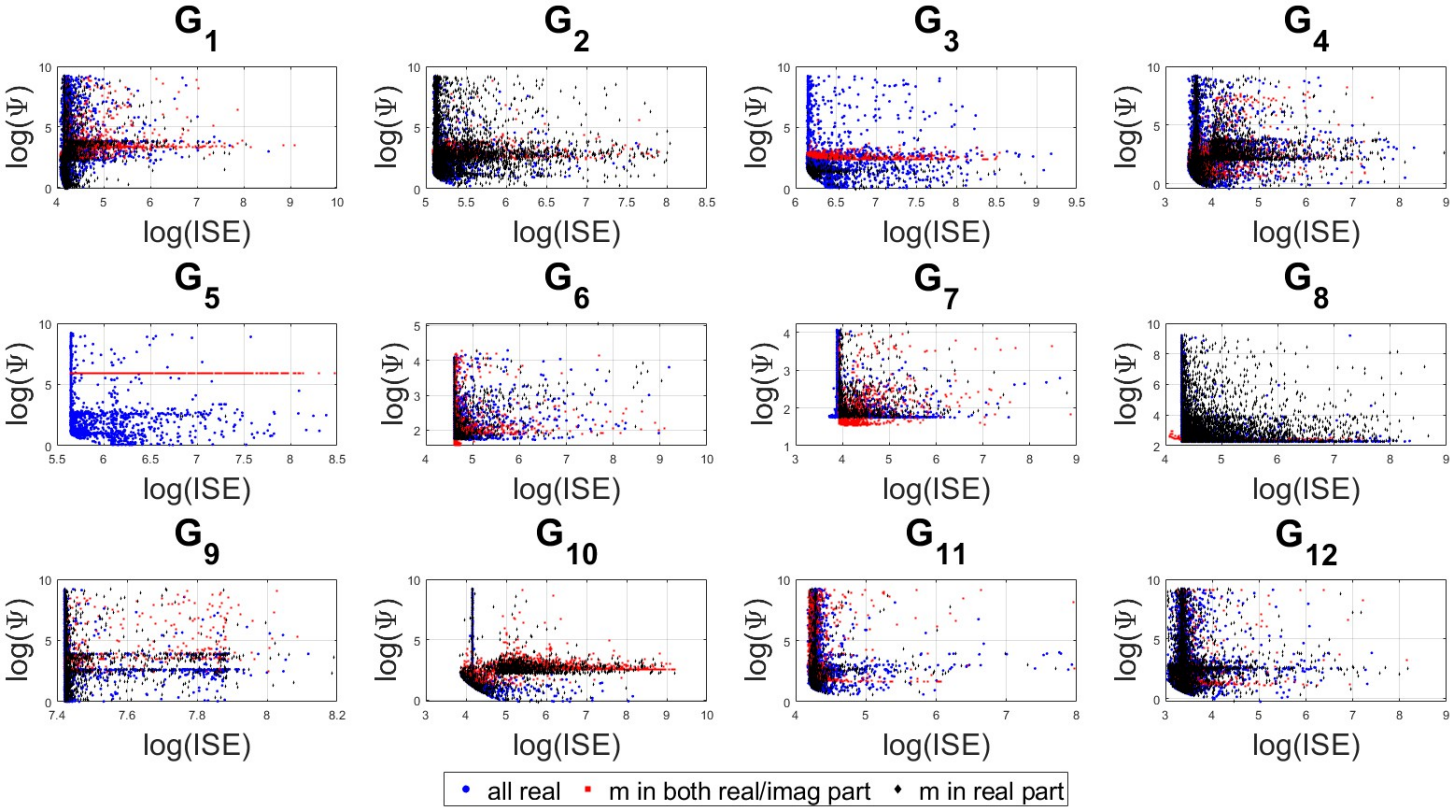
Trade-off between both the objectives with a threshold ISE< 10^4^.

Fig 13 shows the scatter plots of the visited points in the bi-objective space of hybrid cost function (39) in the log-scale. The dense regions show the samples converge to a specific location since we put more emphasis on a small Ψ over small ISE. This is because finding stabilizable region satisfying the dominant pole placement condition was the primary objective of this research. Although only the best compromise solution of the controller parameter has been used to show the time and frequency response of the closed loop control systems, the bi-objective scatter plots gives a proxy for the approximate bivariate density in the two objective function space between the three alternative controller expressions.

## 5 Discussions

This paper proposes a dominant pole placement based filtered PID controller design methodology for handling a general class of SOPTDZ process which has more complex dynamics, as compared to the first order plus time delay with zero (FOPTDZ) [38] and SOPTD models [23–25]. The proposed controller ensures robust stability and performance where the robust stabilizable region has been obtained in the controller and design parameter space, using random search and optimization technique. Here, the transcendental exponential delay term is converted to a finite number of poles by appropriate choice of the sampling time. This is how it can avoid finite order approximations e.g. Pade to handle quasi-polynomial characteristic equations due to the presence of the delay terms. Also, Pade approximation may affect the robust stability and performance since it is not unique when designing the controller for SOPTDZ system. In this paper, the SOPTDZ system and filtered PID controller are discretized with a specified sampling time (*T*_*s*_) using pole-zero matching method. In order to obtain analytical expressions of three distinct filterd PID controller for three different non-dominant pole types, the coefficient matching method has been used. Therefore, the proposed method does not require state space model where Lyapunov based approach is used for stability analysis. The obtained three distinct analytical expressions are implicit in nature which are solved simultaneously. By minimizing the set-point tracking performance measure i.e. ISE criterion [23–25, 38], PSO based random search approach is used. However, it is well known that a controller is said to be *fragile* if small perturbations in the controller parameters cause the closed loop system with a fixed (nominal) plant to become unstable. This means that while designing a controller for a fixed plant, it must be ensured that slight changes to the controller parameters would not destabilize the closed loop systems [59]. This controller is called *non-fragile*. Utilizing the concept of robustness in terms of stability regions proposed in [59] for designing non-fragile controller in the controller space, here, we have used set of all stabilizing filtered PID controller gains in order to obtain the non-fragile filtered PID controller in the controller parameter space. Moreover, in order to determine the optimum compromise solution within a range of filtered PID controller gains vs. fulfillment of the polynomial criteria, we have chosen the objective function (39). Hence, the obtained stability region is an approximate region indicating the degree of robust stability. This is a new approach and not an incremental performance comparison with other existing methods and also this method does not depend on the process model. Although, this paper proposes a novel way to find approximate stabilizable region to ensure robust stability and performance of a general class of SOPTDZ processes by designing dominant pole placement based filtered PID controllers, there are also some limitation as follows:

- Conditions (39) may not be satisfied equally well like minimizing the ISE criterion for all complex SOPTDZ processes.
- The proposed methodology is computationally expensive, especially for many particles in the PSO optimizer.
- The proposed methodology is based on the idea of dominant pole placement where the effect of closed loop zeros has not been considered and will be explored in the future.

## 6 Conclusions

For handling SOPTDZ processes, a novel filtered PID controller design based on dominant pole placement method has been established in this paper. For designing the filtered PID controller using the coefficient matching technique in discrete time with the pole-zero matching discretization method and a chosen sampling time, the various non-dominant poles, e.g. all real and all complex conjugates with three various types, are taken into consideration depending on the pole placement parameter (*m*) position. Three distinct classes of non-dominant poles are given as a set of simultaneous nonlinear implicit equations. From these equations, three different filtered PID controller expressions are derived. Using a PSO-based random search method, they were solved by minimizing a hybrid objective function that was composed of the weighted sum of the residuals from the dominant pole placement criteria and the ISE criterion within a specified range of design parameters and controller parameters. In order to solve the infinite dimensional problem caused by the time delay, the best filtered PID controller parameters are first designed in the discrete time (*z*-domain). These parameters are then mapped back onto the continuous time domain (*s*-domain), making the control system design appear to have been done directly in the continuous time domain. In order to demonstrate the efficacy and generalizability of the suggested method, 12 SOPTDZ test-bench plants with different open loop dynamics are taken into consideration, including stable, integrating, and unstable with both minimum and non-minimum phase zeros.

Although the stabilizing regions provide as a general reference for how resistant the control systems are to model uncertainty, we do not explicitly take them into account when designing the controller. This work can be further extended by including more control objectives like the disturbance rejection responses along with the set-point tracking and extending with delay margins for uncertain systems. Currently the stability region has been discovered using the set-point tracking based ISE criterion. However, for the disturbance input the response may be different which can be used within a multi-objective optimization framework such as [48, 49, 60–64], while keeping the analytical dominant pole placement conditions same. Also, the parameters of the plant including delay are considered to be fixed while searching for the range of PID controller gains. In future, uncertain, noisy, and stochastic systems can be considered along with the use of probabilistic sampling methods for more accurate and efficient approximation of the stabilizable regions.

## References

[1] Bhattacharyya S. P., Datta A., Keel L. H., Linear control theory: structure, robustness, and optimization, CRC Press, 2018.

[2] Astrom K. J., Hang C. C., Lim B., A new smith predictor for controlling a process with an integrator and long dead-time, IEEE Transactions on Automatic Control 39 (2) (1994) 343–345. doi: 10.1109/9.272329

[3] Shamsuzzoha M., Lee M., IMC-PID controller design for improved disturbance rejection of time-delayed processes, Industrial & Engineering Chemistry Research 46 (7) (2007) 2077–2091. doi: 10.1021/ie0612360

[4] Skogestad S., Simple analytic rules for model reduction and PID controller tuning, Journal of Process Control 13 (4) (2003) 291–309. doi: 10.1016/S0959-1524(02)00062-8

[5] F. Alkhafaji, W. Hasan, N. Sulaiman, M. Mohd, M. Sarfraz, S. Karim, A novel PID robotic for speed controller using optimization based tune technique, in: Computational Optimization Techniques and Applications, Vol. 2021, IntechOpen, 2021, pp. 250–254.

[6] Åström K. J., Hägglund T., Revisiting the ziegler–nichols step response method for pid control, Journal of Process Control 14 (6) (2004) 635–650. doi: 10.1016/j.jprocont.2004.01.002

[7] Li H., Niculescu S.-I., Dugard L., Dion J.-M., Robust guaranteed cost control of uncertain linear time-delay systems using dynamic output feedback, Mathematics and Computers in Simulation 45 (3-4) (1998) 349–358. doi: 10.1016/S0378-4754(97)00114-6

[8] Ellis M., Christofides P. D., Economic model predictive control of nonlinear time-delay systems: Closed-loop stability and delay compensation, AIChE Journal 61 (12) (2015) 4152–4165. doi: 10.1002/aic.14964

[9] Niculescu S.-I., H_∞_ memoryless control with an *α*-stability constraint for time-delay systems: an LMI approach, IEEE Transactions on Automatic Control 43 (5) (1998) 739–743. doi: 10.1109/9.668850

[10] Fridman E., Shaked U., An improved stabilization method for linear time-delay systems, IEEE Transactions on Automatic Control 47 (11) (2002) 1931–1937. doi: 10.1109/TAC.2002.804462

[11] Ivănescu D., Lozano R., Niculescu S.-I., On closed-loop stability for mechanical systems with input delays, IFAC Proceedings Volumes 34 (23) (2001) 43–48. doi: 10.1016/S1474-6670(17)32863-X

[12] K. J. Åström, T. Hägglund, PID controllers: theory, design, and tuning, Vol. 2, Instrument society of America Research Triangle Park, NC, 1995.

[13] Onat C., A new design method for PI–PD control of unstable processes with dead time, ISA Transactions 84 (2019) 69–81. doi: 10.1016/j.isatra.2018.08.029 30318365

[14] Tan N., Atherton D. P., Design of stabilizing PI and PID controllers, International Journal of Systems Science 37 (8) (2006) 543–554. doi: 10.1080/00207720600783785

[15] Almodaresi E., Bozorg M., Taghirad H. D., Stability domains of the delay and PID coefficients for general time-delay systems, International Journal of Control 89 (4) (2016) 783–792. doi: 10.1080/00207179.2015.1099166

[16] Silva G. J., Datta A., Bhattacharyya S. P., New results on the synthesis of PID controllers, IEEE Transactions on Automatic Control 47 (2) (2002) 241–252. doi: 10.1109/9.983352

[17] Saeki M., Properties of stabilizing PID gain set in parameter space, IEEE Transactions on Automatic Control 52 (9) (2007) 1710–1715. doi: 10.1109/TAC.2007.904285

[18] Silva G. J., Datta A., Bhattacharyya S. P., PID controllers for time-delay systems, Springer Science & Business Media, 2007.

[19] Shafiei Z., Shenton A., Tuning of PID-type controllers for stable and unstable systems with time delay, Automatica 30 (10) (1994) 1609–1615. doi: 10.1016/0005-1098(94)90100-7

[20] Keel L. H., Bhattacharyya S. P., Controller synthesis free of analytical models: Three term controllers, IEEE Transactions on Automatic Control 53 (6) (2008) 1353–1369. doi: 10.1109/TAC.2008.925810

[21] Wang D.-J., Further results on the synthesis of PID controllers, IEEE Transactions on Automatic Control 52 (6) (2007) 1127–1132. doi: 10.1109/TAC.2007.899045

[22] Wang D.-J., A PID controller set of guaranteeing stability and gain and phase margins for time-delay systems, Journal of Process Control 22 (7) (2012) 1298–1306. doi: 10.1016/j.jprocont.2012.05.019

[23] Das S., Halder K., Gupta A., Performance analysis of robust stable PID controllers using dominant pole placement for soptd process models, Knowledge-Based Systems 146 (2018) 12–43. doi: 10.1016/j.knosys.2018.01.030

[24] Das S., Halder K., Gupta A., Delay handling method in dominant pole placement based PID controller design, IEEE Transactions on Industrial Informatics 16 (2) (2019) 980–991. doi: 10.1109/TII.2019.2918252

[25] Halder K., Das S., Gupta A., Time delay handling in dominant pole placement with PID controllers to obtain stability regions using random sampling, International Journal of Control 94 (12) (2021) 3384–3405. doi: 10.1080/00207179.2020.1764110

[26] Wang H., Han Q.-L., Liu J., He D., Discrete-time filter proportional–integral–derivative controller design for linear time-invariant systems, Automatica 116 (2020) 108918. doi: 10.1016/j.automatica.2020.108918

[27] Martelli G., Stability of PID-controlled second-order time-delay feedback systems, Automatica 45 (11) (2009) 2718–2722. doi: 10.1016/j.automatica.2009.05.031

[28] C. Méndez-Barrios, S.-I. Niculescu, C.-I. Morarescu, K. Gu, On the fragility of PI controllers for time-delay SISO systems, in: 2008 16th Mediterranean Conference on Control and Automation, IEEE, 2008, pp. 529–534.

[29] I.-C. Morărescu, C.-F. Méndez-Barrios, S.-I. Niculescu, K. Gu, Stability crossing boundaries and fragility characterization of PID controllers for SISO systems with I/O delays, in: Proceedings of the 2011 American Control Conference, IEEE, 2011, pp. 4988–4993.

[30] Tan N., Computation of stabilizing PI and PID controllers for processes with time delay, ISA Transactions 44 (2) (2005) 213–223. doi: 10.1016/S0019-0578(07)90000-2 15868860

[31] Wang Q.-G., Zhang Z., Astrom K. J., Zhang Y., Zhang Y., Guaranteed dominant pole placement with PID controllers, IFAC Proceedings Volumes 41 (2) (2008) 5842–5845. doi: 10.3182/20080706-5-KR-1001.00985

[32] Halder K., Das S., Gupta A., Transformation of LQR weights for discretization invariant performance of PI/PID dominant pole placement controllers, Robotica 38 (2) (2020) 271–298. doi: 10.1017/S0263574719000596

[33] Saha S., Das S., Das S., Gupta A., A conformal mapping based fractional order approach for sub-optimal tuning of PID controllers with guaranteed dominant pole placement, Communications in Nonlinear Science and Numerical Simulation 17 (9) (2012) 3628–3642. doi: 10.1016/j.cnsns.2012.01.007

[34] Wang H., Liu J., Zhang Y., New results on eigenvalue distribution and controller design for time delay systems, IEEE Transactions on Automatic Control 62 (6) (2016) 2886–2901. doi: 10.1109/TAC.2016.2637002

[35] Dincel E., Söylemez M. T., Digital PI-PD controller design for arbitrary order systems: Dominant pole placement approach, ISA Transactions 79 (2018) 189–201. doi: 10.1016/j.isatra.2018.04.009 29729973

[36] Wang Q.-G., Liu M., Hang C. C., Approximate pole placement with dominance for continuous delay systems by PID controllers, The Canadian Journal of Chemical Engineering 85 (4) (2007) 549–557. doi: 10.1002/cjce.5450850416

[37] O’Dwyer A., Handbook of PI and PID controller tuning rules, Imperial College Press, 2009.

[38] Das S., Halder K., Stabilizing region in dominant pole placement based discrete time PID control of delayed lead processes using random sampling, Chaos, Solitons & Fractals 165 (2022) 112873. doi: 10.1016/j.chaos.2022.112873

[39] Franklin G. F., Powell J. D., Workman M. L., et al., Digital control of dynamic systems, Vol. 3, Addison-Wesley Reading, MA, 1998.

[40] Ogata K., Discrete-time control systems, Prentice Hall Englewood Cliffs, NJ, 1995.

[41] Kiong T. K., Qing-Guo W., Chieh H. C., Hägglund T. J., Advances in PID control, Springer, 1999.

[42] K. J. Åström, T. Hägglund, Advanced PID control, ISA-The Instrumentation, Systems, and Automation Society, Research Triangle Park, NC, 2006.

[43] Bellen A., Guglielmi N., Ruehli A. E., Methods for linear systems of circuit delay differential equations of neutral type, IEEE Transactions on Circuits and Systems I: Fundamental Theory and Applications 46 (1) (1999) 212–215. doi: 10.1109/81.739268

[44] Bellen A., Guglielmi N., Zennaro M., Numerical stability of nonlinear delay differential equations of neutral type, Journal of Computational and Applied Mathematics 125 (1-2) (2000) 251–263. doi: 10.1016/S0377-0427(00)00471-4

[45] Shampine L. F., Dissipative approximations to neutral DDEs, Applied Mathematics and Computation 203 (2) (2008) 641–648. doi: 10.1016/j.amc.2008.05.010

[46] J. Doyle, B. Francis, A. Tannenbaum, Feedback control theory. courier corporation (2013).

[47] Herreros A., Baeyens E., Peran J. R., Design of PID-type controllers using multiobjective genetic algorithms, ISA Transactions 41 (4) (2002) 457–472. doi: 10.1016/S0019-0578(07)60102-5 12398277

[48] Pan I., Das S., Frequency domain design of fractional order PID controller for AVR system using chaotic multi-objective optimization, International Journal of Electrical Power & Energy Systems 51 (2013) 106–118. doi: 10.1016/j.ijepes.2013.02.021

[49] Das S., Pan I., On the mixed H_2_/H_∞_ loop-shaping tradeoffs in fractional-order control of the AVR system, IEEE Transactions on Industrial Informatics 10 (4) (2014) 1982–1991. doi: 10.1109/TII.2014.2322812

[50] Das S., Saha S., Das S., Gupta A., On the selection of tuning methodology of FOPID controllers for the control of higher order processes, ISA Transactions 50 (3) (2011) 376–388. doi: 10.1016/j.isatra.2011.02.003 21420085

[51] Zamani M., Karimi-Ghartemani M., Sadati N., Parniani M., Design of a fractional order PID controller for an AVR using particle swarm optimization, Control Engineering Practice 17 (12) (2009) 1380–1387. doi: 10.1016/j.conengprac.2009.07.005

[52] Shamsuzzoha M., Skogestad S., The setpoint overshoot method: A simple and fast closed-loop approach for PID tuning, Journal of Process Control 20 (10) (2010) 1220–1234. doi: 10.1016/j.jprocont.2010.08.003

[53] Pandey S., Majhi S., Relay-based identification scheme for processes with non-minimum phase and time delay, IET Control Theory & Applications 13 (15) (2019) 2507–2519. doi: 10.1049/iet-cta.2018.6170

[54] Majhi S., et al., Identification of non-minimum phase processes with time delay in the presence of measurement noise, ISA Transactions 57 (2015) 245–253. doi: 10.1016/j.isatra.2015.03.015 25890691

[55] Shamsuzzoha M., Skliar M., Lee M., Design of IMC filter for PID control strategy of open-loop unstable processes with time delay, Asia-Pacific Journal of Chemical Engineering 7 (1) (2012) 93–110. doi: 10.1002/apj.497

[56] Anil C., Sree R. P., Tuning of PID controllers for integrating systems using direct synthesis method, ISA Transactions 57 (2015) 211–219. doi: 10.1016/j.isatra.2015.03.002 25800952

[57] Rao A. S., Chidambaram M., Control of unstable processes with two RHP poles, a zero and time delay, Asia-Pacific Journal of Chemical Engineering 1 (1-2) (2006) 63–69. doi: 10.1002/apj.8

[58] Sree R. P., Chidambaram M., Simple method of calculating set point weighting parameter for unstable systems with a zero, Computers & Chemical Engineering 28 (11) (2004) 2433–2437. doi: 10.1016/j.compchemeng.2004.04.005

[59] Ho M.-T., Datta A., Bhattacharyya S., Robust and non-fragile PID controller design, International Journal of Robust and Nonlinear Control 11 (7) (2001) 681–708. doi: 10.1002/rnc.618

[60] Das S., Pan I., Das S., Multi-objective LQR with optimum weight selection to design fopid controllers for delayed fractional order processes, ISA Transactions 58 (2015) 35–49. doi: 10.1016/j.isatra.2015.06.002 26096954

[61] Das S., Das S., Pan I., Multi-objective optimization framework for networked predictive controller design, ISA Transactions 52 (1) (2013) 56–77. doi: 10.1016/j.isatra.2012.09.004 23040638

[62] Pan I., Das S., Fractional-order load-frequency control of interconnected power systems using chaotic multi-objective optimization, Applied Soft Computing 29 (2015) 328–344. doi: 10.1016/j.asoc.2014.12.032

[63] Pan I., Das S., Das S., Multi-objective active control policy design for commensurate and incommensurate fractional order chaotic financial systems, Applied Mathematical Modelling 39 (2) (2015) 500–514. doi: 10.1016/j.apm.2014.06.005

[64] Pan I., Das S., Chaotic multi-objective optimization based design of fractional order PI^λ^D^*μ*^ controller in AVR system, International Journal of Electrical Power & Energy Systems 43 (1) (2012) 393–407. doi: 10.1016/j.ijepes.2012.06.034

